# Accurate identification of RNA editing sites from primitive sequence with deep neural networks

**DOI:** 10.1038/s41598-018-24298-y

**Published:** 2018-04-16

**Authors:** Zhangyi Ouyang, Feng Liu, Chenghui Zhao, Chao Ren, Gaole An, Chuan Mei, Xiaochen Bo, Wenjie Shu

**Affiliations:** 1 Department of Biotechnology, Beijing Institute of Radiation Medicine, Beijing, 100850 China; 2 Department of information, the 188th hospital of ChaoZhou, ChaoZhou, 521000 China; 3 Department of medical services, the 188th hospital of ChaoZhou, ChaoZhou, 521000 China

## Abstract

RNA editing is a post-transcriptional RNA sequence alteration. Current methods have identified editing sites and facilitated research but require sufficient genomic annotations and prior-knowledge-based filtering steps, resulting in a cumbersome, time-consuming identification process. Moreover, these methods have limited generalizability and applicability in species with insufficient genomic annotations or in conditions of limited prior knowledge. We developed DeepRed, a deep learning-based method that identifies RNA editing from primitive RNA sequences without prior-knowledge-based filtering steps or genomic annotations. DeepRed achieved 98.1% and 97.9% area under the curve (AUC) in training and test sets, respectively. We further validated DeepRed using experimentally verified U87 cell RNA-seq data, achieving 97.9% positive predictive value (PPV). We demonstrated that DeepRed offers better prediction accuracy and computational efficiency than current methods with large-scale, mass RNA-seq data. We used DeepRed to assess the impact of multiple factors on editing identification with RNA-seq data from the Association of Biomolecular Resource Facilities and Sequencing Quality Control projects. We explored developmental RNA editing pattern changes during human early embryogenesis and evolutionary patterns in *Drosophila* species and the primate lineage using DeepRed. Our work illustrates DeepRed’s state-of-the-art performance; it may decipher the hidden principles behind RNA editing, making editing detection convenient and effective.

## Introduction

RNA editing is a post-transcriptional modification^1^ that makes a mature RNA sequence different from its template DNA sequence by inserting, deleting, or substituting bases. RNA editing plays a critical role in many biological processes, including neuronal function^2,3^, cancer development^4–6^, embryogenesis^7,8^, and immune response^9^. A-to-I editing, the most common type of RNA editing in metazoans^10^, refers to the process of adenosine (A) deamination to inosine (I); I is then decoded as guanosine (G) in translation, which is mediated by members of the double-strand RNA-specific adenosine deaminase act on RNA (ADAR) family^11^. A-to-I editing can expand the transcriptomic and proteomic diversity by generating RNA mutations and altering gene regulation. It is evolutionarily conserved and is hypothesized to facilitate adaption in metazoans^11–13^. Recoding A-to-I editing sites have been shown to be essential to cellular function and development by inducing amino acid changes in coding regions^14,15^. Previous studies showed that the abnormality of A-to-I editing is closely related to human diseases, especially in central nervous system (CNS) diseases and cancers^16–20^. Abundant A-to-I editing sites in CNS affect receptors and ion channels expression^21^. The editing level of A-to-I editing is significantly elevated in various cancer tissues^22^. The prevalence and biological function of A-to-I editing have been illuminated widely. Besides, there are still some non A-to-I editing sites that being verified to be functionally meaningful^23–25^. Therefore, it is vital to identify RNA editing sites accurately.

The emergence of next-generation sequencing technologies has greatly facilitated the identification of RNA editing sites by using high-throughput sequencing based methods^26^. The development of powerful computational pipelines has made the study of RNA editing using RNA-seq data pervasive. In 2013, Li developed the first methods, including the separate samples and pooled samples methods, to identify RNA editing sites using RNA sequencing data alone without the need for matched genome sequencing^27^. After that, a prediction method was established to accurately predict constitutive RNA editing sites by using new parameters namely Hits Per Billion-mapped-bases (HPB) and Potential SNP Score (PPS)^28^. GIREMI is a software package that utilizes allelic linkage and generalized linear models to distinguish between RNA editing sites and genetic variants in a single RNA-seq sample^29^. RNAEditor provides an easy-to-use tool to identify RNA editing events and developed a clustering algorithm to find editing islands^30^. These methods, which use RNA-seq data alone with prior-knowledge-based filtering, have greatly facilitated RNA editing detection and effectively use public transcriptomic sequencing datasets without available DNA sequencing data. However, the filtering steps based on prior knowledge and public genome annotations, such as Alu repeats, genomic duplications, and pseudogenes, are cumbersome and time consuming. Moreover, arbitrary and artificial combinations of filtering steps may result in different candidate RNA editing sites. Furthermore, these methods suffer from limited generalization and applicability to other species due to insufficient genomic annotations and prior knowledge. For example, the absence of Alu repeat annotations in the *Drosophila* genome makes the identification of RNA editing inefficient, whereas the deficiency of single nucleotide polymorphism (SNP) information in some species makes the GIREMI and RNAEditor non-applicable. In 2016, two user-friendly web servers, called PAI and iRNA-AI, were established to identify A-to-I editing from RNA sequence information alone^31,32^. These two methods both employed support vector machine as classifier. PAI incorporated six RNA physiochemical properties and global sequence order information to identify A-to-I editing sites in D. melanogaster^31^. iRNA-AI incorporated the chemical properties of nucleotides and their sliding occurrence density distribution to identify human A-to-I editing sites^32^. These two methods provide insights into the identification of RNA editing sites based on machine learning method.

In this study, we present a deep learning-based method called DeepRed that accurately identifies RNA editing by learning and summarizing essential features from the surrounding primitive sequence of candidate SNVs directly without requiring prior-knowledge-based filtering steps. DeepRed achieved 98.1% and 97.9% area under the curve (AUC) in a training set and test set, respectively, indicating that DeepRed manifests good generalization ability. Then, we validated our DeepRed method using independently experimentally verified RNA-seq data from a U87 cell line^33^ and achieved 97.9% positive predictive value (PPV), demonstrating the high prediction accuracy of our DeepRed method. Additionally, using experimentally verified RNA-seq data in K562 and HepG2 cell lines^34^, DeepRed achieved consistently superior performance in comparison with current state-of-the-art methods, including separate samples, GIREMI, RNAEditor and Prediction methods. Further performance assessment of DeepRed in mass RNA-seq data from large-scale RNA-seq studies demonstrated that DeepRed exhibited superior performance with the highest A-to-I ratio, lowest false discovery rate, and highest computational efficiency relative to these state-of-the-art methods.

Moreover, we used RNA-seq data from the Association of Biomolecular Resource Facilities (ABRF)^35^ and Sequencing Quality Control projects (SEQC)^36^ projects to assess the impact of a series of factors, including flanking bases of RNA editing sites, library preparation, RNA degradation, sequence depth, laboratory, read mapping, and variant calling, on the identification of RNA editing. We found that the identified number of RNA editing sites between intact and degraded RNA and different library methods, laboratories, sequence depths, or combinations of read mapping and variant calling methods varied largely. However, the prediction accuracy of RNA editing identified in these conditions, except for sequence depth, are roughly consistent. The prediction accuracy of RNA editing sites may deteriorate at depths less than 15 million mapped reads, and 15 million mapped reads can ensure adequate accuracy of RNA editing identification. The reproducibility of RNA editing sites in these experimental conditions are relatively low, whereas the reproducibility of RNA editing sites identified between different read mapping and variant calling methods is much higher.

Finally, we applied DeepRed to explore the developmental pattern of RNA editing changes during human early embryogenesis and the evolutionary pattern of RNA editing in the primate lineage and *Drosophila* species. We discovered the stage-specific change pattern of RNA editing at 8-cell, morula and late blastocyst stages during human early embryogenesis and the evolutionary conservation of RNA editing between close lineages. Together, our work illustrates the superior performance of DeepRed at identifying RNA editing from RNA sequences without genomic annotations or prior-knowledge-based filtering steps. DeepRed will make the detection of RNA editing convenient and effective and will facilitate future studies on RNA editing.

## Results

### Accurate identification of RNA editing with DeepRed

**Figure 1:**
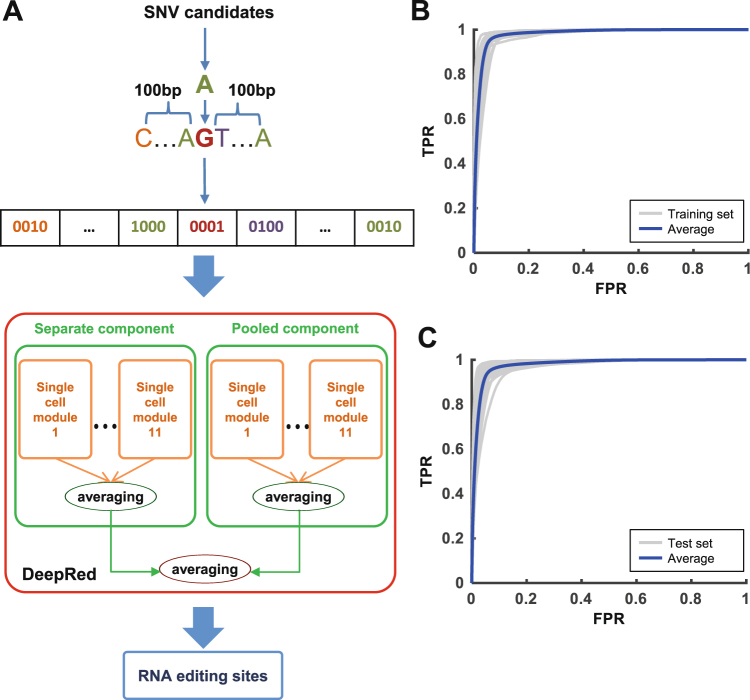
The architecture and performance of DeepRed. (**A**) The hybrid framework of DeepRed. DeepRed consists of separate and pooled components to account for the features of RNA editing derived from the separate samples and pooled samples methods, respectively. Each component combines 11 independent single-cell modules together using a simple averaging method. The separate and pooled components are combined by a simple averaging method. The input of DeepRed is a one-hot-encoded sequence of 201 base pairs (bp) centred at the candidate SNV. (**B**) The ROC curves (grey) of DeepRed were achieved in 22 gold standard sets in the training set. The average ROC curve of DeepRed in the training set is presented as a blue curve. (**C**) The ROC curves (grey) of DeepRed were achieved in 42 gold standard sets in the test set. The average ROC curve of DeepRed in the test set is presented as a blue curve.

We developed DeepRed, a deep learning-based hybrid framework integrated with ensemble learning, to precisely and conveniently predict RNA editing sites using RNA-seq data alone. DeepRed learns and summarizes essential features from the surrounding primitive sequence of candidate SNVs directly without requiring complicatedly prior-knowledge-based filtering steps. It could identify all 12 possible types of RNA editing sites, of which A-to-I is the most prevalent mismatch type. Based on the positive and negative sets that we constructed (Fig. S1, Table S1, see “Materials and Methods”), we designed DeepRed to consist of a separate component and a pooled component to account for the features of RNA editing derived from the separate samples and pooled samples methods, respectively, with each component including 11 independent single-cell modules (Fig. 1A). Our DeepRed method used the one-hot-encoded primitive sequence centred at candidate SNVs as an input and calculated a score for each candidate. DeepRed considered SNVs that have larger scores than the cut-off to be RNA editing sites.

For the basic module of DeepRed, we first trained the single-cell module using the gold standard set in Sknshra cells to optimize its network structure using modified 5-fold cross-validation (Figs 1A and S2) (see “Materials and Methods”). We obtained the optimized structure of the single-cell module, which combined two ensemble DNNs with respective input scales of 101 bp and 41 bp. Each ensemble DNN consisted of 20 individual DNNs, which were combined using a simple averaging method (Fig. S3A–C). In the simple averaging method, the predicted probabilities from each individual DNNs were averaged to produce a single estimation. The bagging-style bootstrapped resampling method was used as a parallel ensemble method to solve the class-imbalanced problem and improve generalization capability (Fig. S4, see “Supplemental materials”). We evaluated the performance of the single-cell module on independent U87 data^33^ using modified 5-fold cross-validation (Fig. S5) (see “Materials and Methods”). We achieved 93.3% AUC and 86.6% GM on U87 data, suggesting that the single-cell module illustrates superior performance (Fig. S5).

Then, we used the optimized structure of the single-cell module as the basic unit of the separate and pooled components of DeepRed. For the separate component of DeepRed, we used 11 gold standard sets in the separate training set to train the respective 11 single-cell modules and used the simple averaging method to combine these modules (Fig. S6A, see “Supplemental materials”). In the same way, the pooled component of DeepRed was trained using the 11 gold standard sets in the pooled training set (Fig. S6A). Finally, we combined the separate and pooled components as the final ensemble classifier of DeepRed using a simple averaging method (Figs S6B and S7). We assessed the performance of DeepRed by first evaluating it with the separate and pooled training sets. DeepRed achieved an average of 98.1% AUC and 88.4% area under the precision-recall curve (AUCPR) on the training sets, illustrating its superior performance in RNA editing detection (Figs 1B and S8A). We selected two other independent separate and pooled test sets, each of which contained 21 test sets covering various cells/tissues that were not used in the training procedure, to avoid possible overestimation of DeepRed’s performance on training sets. Again, DeepRed achieved an average of 97.9% AUC and 87.0% AUCPR in the test sets (Figs 1C and S8B). Assessment of the hybrid framework integrating deep learning with ensemble learning on the training and test sets demonstrated that the hybrid structure indeed improved RNA editing prediction performance (Fig. S9 and Table S2, see “Materials and Methods”). Taken together, these results demonstrated the superior performance and generalization ability of our DeepRed method.

### Validation of DeepRed using experimentally verified data

We validated whether the editing sites identified with DeepRed were bona fide editing events by identifying RNA editing sites using an independent U87 test sample from ENCODE and comparing the sites to those identified from experimental samples of wild-type and knock-down U87 (Figs S10 and S11, Table S3, see “Materials and Methods”). For comparison, we identified RNA editing sites using four state-of-the-art methods, the separate samples method^27^, Prediction method^28^, the GIREMI method^29^, RNAEditor^30^, and set suitable cut-offs for DeepRed to generate similar numbers of identified RNA editing sites to the four methods. Our DeepRed method identified 167 RNA editing sites from the 12,609 candidate SNVs of the U87 test sample. When we compared these sites to those identified from U87 experimental samples, 48 RNA editing sites were experimentally verified; 47 were verified to be true RNA editing sites and 1 was verified to be a false RNA editing site. Thus, our DeepRed method achieved the 97.9% (47/48) PPV.

In addition, we compared the performance of our DeepRed method with the four methods using eight performance indicators (Table 1). Based on the performance indicators (accuracy, specificity, GM, positive predict value, F1-score, and misclassification rate), DeepRed consistently performed better than the four methods. Based on sensitivity, separate samples method was ranked first followed by DeepRed, RNAEditor, Prediction and GIREMI. In terms of validation rate, RNAEditor has best result followed by separate samples method, DeepRed, GIREMI and Prediction method. We ranked the performance of the five methods based on these eight performance indicators, and averaged the ranked positions of each of the three methods in all eight tests (Table S4). This analysis revealed that our DeepRed method achieved higher accuracy than the other four state-of-the-art methods and that it showed superior ability in predicting positives and recognizing negatives simultaneously. Moreover, we performed the same validation analysis on experimentally verified RNA-seq data in K562 and HepG2 cells^34^ (Table S3). This analysis again illustrated the performance superiority of our DeepRed method relative to the four state-of-the-art methods (Tables S5 and S6). Together, our results revealed that our DeepRed method could accurately identify RNA editing with superior performance without using genome annotations or prior knowledge-based filtering steps.

**Table 1:** Performance comparison of DeepRed with separate samples, GIREMI, RNAEditor and Prediction methods on U87 data.

Method	Accuracy	Sensitivity	Specificity	GM	Positive predict value	F1 score	Validation rate	Misclassification rate
DeepRed	93.23%	79.66%	99.25%	88.92%	97.92%	88.38%	29.01%	0.62%
Separate	76.04%	83.05%	72.93%	77.83%	57.65%	77.66%	29.34%	21.56%
GIREMI	70.31%	6.78%	98.50%	25.84%	66.67%	12.69%	14.81%	7.41%
RNAEditor	89.58%	69.49%	98.50%	82.73%	95.35%	81.49%	40.59%	1.98%
Prediction	63.54%	15.25%	84.96%	36.00%	31.03%	25.86%	7.83%	17.39%

### Performance assessment across methods using mass RNA-seq data

**Figure 2:**
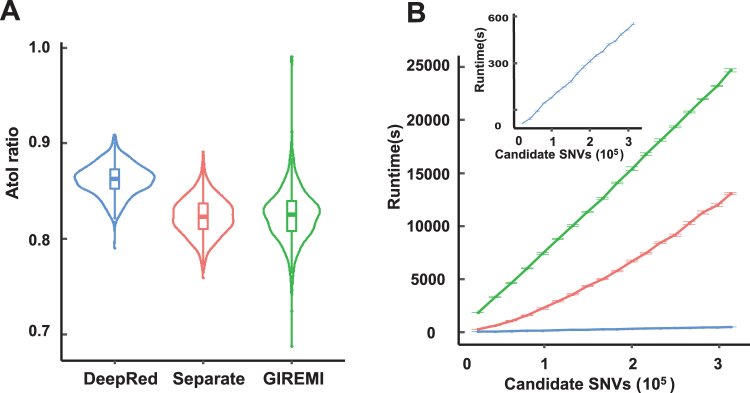
Performance comparison of DeepRed with separate samples method and GIREMI method. (**A**) Violin plot of the A-to-I ratios identified by DeepRed, separate samples method, and GIREMI method for each individual in the Geuvadis dataset. The first, second (median), and third quartiles are illustrated in box-plot style. (**B**) The relationship between the candidate SNVs and runtime of RNA editing sites identified with DeepRed (blue), separate samples method (red), and GIREMI method (green). The insert plot represents the relationship between the candidate SNVs and runtime of RNA editing identified with DeepRed. Runtime refers to the time spent identifying RNA editing sites from candidate SNVs. Error bar represents the standard error of runtime across ten down sampling samples.

To further assess the performance of our DeepRed, we performed a comprehensive performance comparison of our DeepRed with other three state-of-the-art methods: separate samples, pooled samples, and GIREMI methods. We applied these methods to identify RNA editing sites using massive RNA-seq data of lymphoblastoid cells in the Geuvadis Project^37^ (Table S7). Considering that GIREMI had a strict filtering procedure that required each editing site had a total read coverage of >=5 and a supporting read coverage of >=3, we conducted the same filter of read coverage depth on other three methods to carry out a fair comparison (Table 2, see “Materials and Methods”). As expected, the pooled method predicted editing sites five times more than those of other methods, since it pooled together RNA-seq alignments from different individuals and achieved a much higher read coverage. Assuming all non A-to-I mismatches are false and the error rate for all 12 mismatch types is equal, DeepRed achieved the lowest FDR of only 2.4% and the highest A-to-I ratio of 79.1% across 462 individual samples. In addition, DeepRed achieved the highest median A-to-I ratio, the lowest variance of A-to-I ratio, and the lowest median FDR in 462 individuals (Figs 2A and S12A). Our comparison analysis indicated that DeepRed manifested the highest accuracy in RNA editing site identification using RNA-seq data alone. In addition, DeepRed consistently performed better than all other methods even if we did not conduct read coverage filtering (Table S8). Furthermore, we performed the same performance comparison analysis among these methods using mass RNA-seq data from large-scale RNA-seq studies, including ENCODE^38^, Roadmap Epigenomics^39^, and CCLE^40^ projects. Our DeepRed again achieved consistently superior performance with the highest A-to-I ratio and the lowest FDR (Fig. S13A–F).

**Table 2:** A-to-I ratio of DeepRed, separate samples, pooled samples and GIREMI methods on Geuvadis data.

Method	Number of predicted RNA editing sites		A-to-I%	FDR
	All	A-to-I		
DeepRed	100,116	79,172	79.08%	2.40%
GIREMI	93,910	62,222	66.26%	4.63%
Separate	146,173	95,986	65.67%	4.75%
Pooled	538,467	372,744	69.22%	4.04%

Next, we compared the overall runtime of DeepRed to that of separate samples and GIREMI methods by down sampling the RNA-seq data of human brain reference from SEQC project (Table S9). The runtime is almost linearly positively related to the sequence depth and the number of SNV candidates for these three methods (Figs 2B and S12B). Our DeepRed takes less than one-tenth of the separate samples method’s runtime, and less than one-third of GIREMI’s runtime to identify RNA editing sites. It is due to that our DeepRed uses only a list of SNVs as inputs and uses none of any prior-knowledge-based filtering steps and genomic annotations. Together, our results convincingly demonstrated the state-of-the-art performance of DeepRed relative to the existing methods.

### Impact of multiple factors on the identification of RNA editing sites

Next, we applied DeepRed to RNA-seq data from the ABRF^35^ and SEQC projects^36^ to assess the impact of a range of factors, including flanking bases of RNA editing sites, library preparation methods, RNA degradation methods, laboratory, sequence depth, read mapping and variant calling methods, on the identification of RNA editing sites (see “Supplemental materials”).

First, to find out which bases in flanking region of RNA editing sites were important for prediction, we used the R package randomForest^41^ based on site importance score namely the mean decrease in Gini index to assess the site importance for prediction. We found that the site importance score decreased with the increasement of distance to the candidate site (Fig. S14). Detailly, the 201-bp sites could be intuitively divided into 3 different regions according to the importance score: highly-important region, namely −2 bp to 2 bp, medianly-important region, namely −25 bp to −2 bp and 2 bp to 25 bp, and lowly-important region, namely −100 bp to −25 bp and 25 bp to 100 bp. The highly-important and medianly-important region namely −25 bp to 25 bp region contributed most to the identification of RNA editing site, which was consistent with previous studies^31,32^. Besides, we could also find out that guanine contributed least at the −1 bp site and contributed most at 1 bp site to the identification of RNA editing site, which was consistent with a previous report that ADARs have a sequence preference for “G” depletion and “G” enrichment at the 5′ and 3′ neighbor nucleotides next to A-to-I editing sites, respectively^27,42^.

**Figure 3:**
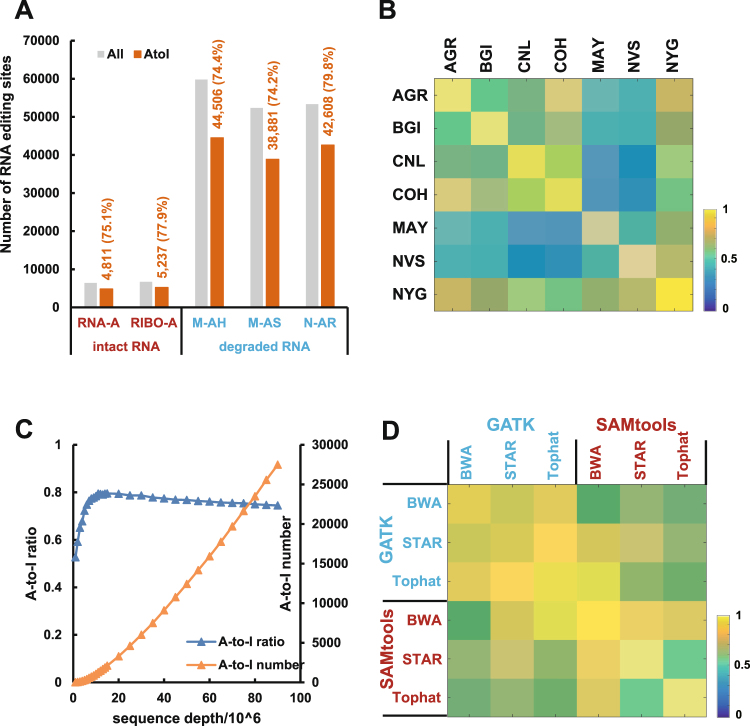
Impact of multiple factors on the identification of RNA editing. (**A**) The number of RNA editing sites identified from intact RNA prepared by different library methods (poly-A-enriched or ribo-depleted) (left) and RNA degradation methods (heat, sonication or RNase-A) (right). The grey and orange bars represent the number of RNA editing sites and number of A-to-I editing sites, respectively. The number of predicted editing sites and A-to-I ratio are listed in orange. RNA-A: poly-A-enriched; RIBO-A: ribo-depleted; M-AH: heat; M-AS: sonication; N-AR: RNase-A. (**B**) The heatmap represents the reproducibility of RNA editing sites identified from different laboratories, including AGR (Australian Genome Research Facility), BGI (Beijing Genomics Institute), CNL (Weill Cornell Medical College), COH (City of Hope), MAY (Mayo Clinic), NVS (Novartis), and NYG (the New York Genome Center). (**C**) The relationship between the sequence depth (mapped reads) and the number of A-to-I editing sites (orange line) and the A-to-I ratio (blue line). The left Y-axis is the A-to-I ratio, whereas the right Y-axis is the number of A-to-I editing sites. Each point represents the average value of 10 repeated analyses at that sequence depth. (**D**) The heatmap represents the reproducibility of RNA editing sites for different read mapping methods (BWA, STAR, and Tophat) and variant calling methods (GATK and SAMtools).

Then, we examined the influence of library preparation and RNA degradation on the detection of RNA editing sites (Table S10). For intact RNA samples, the number of RNA editing sites identified in poly-A enrichment prepared RNA was similar to that in ribo-depleted prepared RNA (6,403 VS 6,719) (Fig. 3A). The ribo-depleted RNA identified 77.94% of A-to-I editing sites, which was slightly higher than that identified in Poly-A RNA (75.14%) (Fig. S15A). For ribo-depletion RNA degraded using heat, sonication or RNase-A, the number of identified RNA editing sites was 59,807, 52,367 and 53,332, and the corresponding A-to-I ratios of RNA editing sites were 74.25%, 74.42%, 79.89%, respectively (Fig. 3A). The RNA degraded by RNase-A showed a relatively higher percentage of A-to-I editing sites than the other degraded methods (Fig. S15A). These results indicated that the different library preparations and degradation methods produced roughly consistent detection accuracy for RNA editing sites. However, the reproducibility across different library preparation methods and degradation methods was much low, ranging from 0.10 to 0.32 (Fig. S15B). Additionally, the number of identified RNA editing sites in the degraded RNA was eight times higher than that of intact RNA with a ribo-depleted library (Fig. 3A). Thus, the use of combined samples from different library preparations or combinations of intact RNA and degraded RNA samples within an experiment should be avoided in the identification of RNA editing sites.

Next, we explore the impact of different laboratories on RNA editing identification (Table S11). We observed that the number of detected RNA editing sites varied by laboratory, ranging from 13,600 to 41,674 (Fig. S16A). However, a similar ratio of A-to-I editing sites indicated the same prediction accuracy between laboratories (Fig. S16B). The reproducibility of identified RNA editing sites between any two laboratories ranged from 23% to 75% (Fig. 3B). The widely fluctuating reproducibility across laboratories suggested that it is inappropriate to directly compare RNA editing sites identified by different laboratories, regardless of other factors.

Besides, we investigated the impact of sequence depth on RNA editing site identification by down sampling human brain RNA-seq data of 16 individuals from the SEQC project (Table S9). As sequencing depth increased, the number of RNA editing sites stably increased. Notably, the A-to-I ratio increased with sequence depths less than 15 million mapped reads, but it slightly dropped with sequence depths more than 15 million mapped reads (Fig. 3C). A consistent result was obtained using the separate samples method (Fig. S17). Our results indicated that prediction accuracy of RNA editing sites may deteriorate at depths of less than 15 million mapped reads and that a depth of 15 million mapped reads can ensure adequate accuracy of RNA editing identification.

Finally, we assessed the impact of different combinations of read mapping and variant calling methods on the identification of RNA editing sites with the same pooled human brain RNA-seq alignment (Table S9). When we chose GATK^43^ as the variant calling method, we identified 17,951, 45,495, and 57,462 RNA editing sites using Tophat^44^, BWA^45^, and STAR^46^ as the mapping method, respectively. When we chose SAMtools as the variant calling method, we identified 21,417, 44,338, and 114,749 RNA editing sites using STAR, Tophat, and BWA as the mapping method, respectively (Fig. S18A). The number of identified RNA editing sites varied largely with different read mapping methods and variant calling methods. However, the A-to-I ratios were all approximately 70% for different read mapping methods and variant calling methods, indicating that the different read mapping methods and variant calling methods had little effect on the identification accuracy of RNA editing sites (Fig. S18B). Further reproducibility analysis demonstrated that when different combinations of read mapping and variant calling methods were used, the reproducibility of the identified RNA editing sites between any two combination methods was relatively high, ranging from 52.29% to 95.36%, except for the combination of STAR and Tophat with SAMtools as the variant calling method (43.79%) (Fig. 3D). Together, our results suggested that the read mapping and variant calling methods showed similarity in identification accuracy but relatively large differences in the number of identified RNA editing sites.

### Application of DeepRed across species

**Figure 4:**
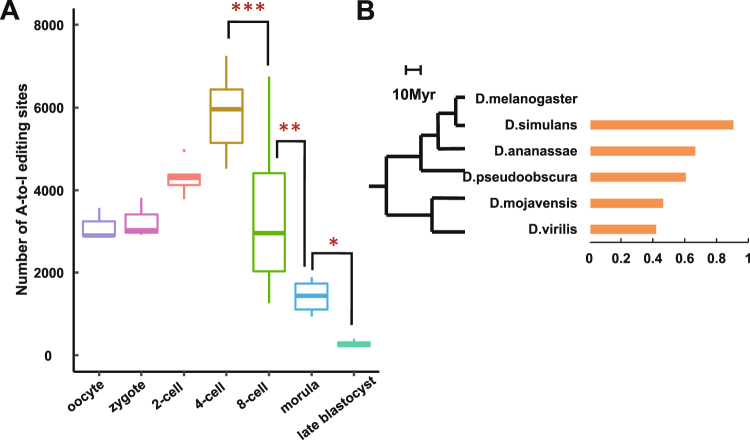
Application of DeepRed. (**A**) The number of RNA editing sites identified with DeepRed during early human embryogenesis. *P < 0.05; **P < 0.01; ***P < 0.001 (Wilcoxon rank sum test). (**B**) The phylogenetic relationships among six analysed *Drosophila* species (left). Myr, million years ago. Ratio of RNA editing sites homologous to *D. melanogaster* in *Drosophila* species (right).

Because of its genome-independent and SNP-free nature, DeepRed can be applied to any RNA-seq dataset without restrictions. Here, we used it to explore the developmental pattern of RNA editing during human early embryogenesis and the evolutionary pattern of RNA editing in *Drosophila* species and the primate lineage. First, we applied DeepRed to identified RNA editing sites using single-cell RNA-seq data from human embryos spanning from the oocyte to late blastocyst stage^47^ (Table S12). On average, we detected 3,119, 3,247, 4,299, 5,833, 3,354, 1,425 and 286 A-to-I editing sites per cell at the oocyte, zygote, 2-cell, 4-cell, 8-cell, morula and late blastocyst stages, respectively. Notably, we observed that the number of A-to-I editing sites reached peak value at 4-cell stage, but it sharply decreased from the 4-cell to 8-cell stage (5,833 vs. 3,354, *P* = 1.5 × 10^−4^, Wilcoxon rank sum test) and continually decreased at the morula (3,354 vs. 1,425, *P* = 8.8 × 10^−3^, Wilcoxon rank sum test) and late blastocyst (1,425 vs. 286, *P* = 0.03, Wilcoxon rank sum test) stages (Fig. 4A). A consistent result was obtained using the separate samples method (Fig. S19). Our results illustrated the stage-specific change patterns of RNA editing during human early embryogenesis.

Next, we explored the evolutionary conservation of RNA editing in *Drosophila* species and the primate lineage (see “Materials and Methods”). We applied DeepRed to RNA-seq data from adult whole bodies of *D. melanogaster*, *D. simulans*, *D. ananassae*, *D. pseudoobscura*, *D. mojavensis*, and *D. virilis*^48^ (Table S13) and identified 6,057, 28,377, 345, 1,107, 531, and 566 A-to-I editing sites, respectively. Further cross-species comparison analysis demonstrated that 25,726, 230, 672, 247, and 238 A-to-I editing sites identified in *D. simulans*, *D. ananassae*, *D. pseudoobscura*, *D. mojavensis*, and *D. virilis*, respectively, were homologous to *D. melanogaster*. It revealed that species closer to *D. melanogaster* had a higher ratio of homologous RNA editing sites (Fig. 4B), indicating that RNA editing events tend to be conserved between related species. Furthermore, we extended our analysis to the primate lineage^49^ (Table S14). We identified 17,250, 14,746, 36,846, and 858 A-to-I editing sites using human, chimpanzee, rhesus macaque and mouse brain RNA-seq data, respectively. We found that 13,346, 28,943, and 93 A-to-I editing sites in chimpanzee, rhesus macaque and mouse brain were homologous to human editing sites. Similarly, chimpanzee, as the closest species to human, had the highest ratio of RNA editing sites that were homologous to human sites (Fig. S20). The results demonstrated evolutionary conservation of A-to-I editing in the primate lineage.

## Discussion

In this study, we developed a hybrid framework, named DeepRed, which integrates deep learning with ensemble learning to accurately identify RNA editing from RNA sequences without prior-knowledge-based filtering steps. DeepRed achieved 98.1% and 97.9% AUC in the training set and test set, respectively, illustrating its strong generalization capability (Figs S3A–C and S8). Further validation of DeepRed performance using independently experimentally verified data in U87, K562, and HepG2 cells revealed that DeepRed exhibited much higher prediction accuracy than current state-of-the-art methods. Further performance assessment of DeepRed in mass RNA-seq data from large-scale RNA-seq studies demonstrated that DeepRed illustrated superior performance with its high A-to-I ratio and low FDR relative to these state-of-the-art methods. Together, our results demonstrated that our DeepRed method has a strong ability to learn and summarize essential features from the surrounding primitive RNA sequences of candidate SNVs. Our DeepRed method can accurately identify RNA editing without prior-knowledge-based filtering steps or genomic annotations. These characteristics facilitate more efficient and generalized identification of RNA editing, especially for the species with insufficient annotation information. Although DeepRed is capable of learning the general features of RNA editing across multiple samples, it may not be sensitive to the recognition of RNA editing sites unique to a single sample.

We applied DeepRed to ABRF and SEQC RNA-seq data collected with multiple library preparation methods and RNA degradation methods from multiple laboratories to assess the impact of multiple factors on the identification of RNA editing. We found that different library preparation methods and RNA degradation methods both showed similarity in identification number and recognition accuracy but had relative large differences in reproducibility. Remarkably, we observed eight times more RNA editing sites in degraded RNA than in intact RNA. This difference was likely because the degradation method stimulated the occurrence of RNA editing or destroyed the integrity of the transcripts. In addition, we found that sequencing depth had a great influence on the accuracy of RNA editing identification. The prediction accuracy of RNA editing identification deteriorates at a lower sequencing depth, suggesting that the identification of RNA editing requires samples with relatively high sequence depths. A sequencing depth of 15 million mapped reads in the human genome ensured adequate accuracy of RNA editing identification. Moreover, we found that the reproducibility of RNA editing sites fluctuated widely across laboratories, even when laboratories used the same protocol to identify RNA editing sites, reminding us to be especially careful when comparing the RNA editing sites of RNA-seq samples from different labs.

The predication accuracy of various read mapping and variant calling methods was similar, but their number of identified editing sites varied greatly. Compared to GATK, SAMtools was a more relaxed variant calling strategy, leading to the prediction of higher numbers of RNA editing sites. As for mapping methods, Tophat was the most stringent mapping approach and identified the fewest RNA editing sites, which enabled more accurate prediction of RNA editing sites; however, this tool may miss some true RNA editing sites that are detected by other mapping methods. We suggested using Tophat-SAMtools or STAR-GATK combination methods to identify RNA editing sites to optimize both prediction accuracy and detection number. Selection of a specific combination of read mapping and variant calling methods is recommended based on the research purpose. In this study, we chose the moderately stringent read mapping method STAR and rigorous variant calling method GATK because they represent the best practice for SNP and indel calling with RNA-seq data.

The exploration of RNA editing in human early embryogenesis showed that the number of A-to-I RNA editing sites changes in a stage-specific manner during human early embryogenesis. We noticed that genome-wide A-to-I RNA editing sites dramatically decreased at the 8-cell, morula and late blastocyst stages, suggesting that the sudden drop of A-to-I RNA editing sites may be associated with a specific regulatory mechanism and may have important biological significance during human early embryogenesis. The dramatically decreased number of RNA editing sites at the 8-cell and morula stages aligned well with that results of a previous study^50^. Although the biological function of stage-specific changes is yet to be discovered, the inflection point of the change pattern at the 8-cell stages suggests that these stages could serve as a good direction for studying regulatory mechanisms during human early embryogenesis. In fact, recent studies on human early embryogenesis have revealed that many important biological events, such as gene expression^47^, X chromosome inactivation (XCI)^51^, and embryonic left-right separation, occur in a stage-specific fashion^52^. We demonstrated that RNA editing could describe the genetic relationship between species through the evolution analysis of RNA editing in *Drosophila* species and the primate lineage. The consistent evolutionary pattern of RNA editing found in *Drosophila* species and the primate lineage agrees well with findings from previous studies^12,53^. These results demonstrated the broad applicability of our DeepRed method and confirmed the reliability of DeepRed from an application point of view.

Taken together, our DeepRed method could decipher the hidden principles behind RNA editing by extracting and learning features from the raw sequences directly and predicted RNA editing sites accurately without prior-knowledge-based filtering steps or genome annotations. Our DeepRed method will make the detection of RNA editing convenient and effective and will facilitate the study of RNA editing.

## Methods

### Construction of positive and negative sets

Considering that no gold standard set of RNA editing sites has been experimentally verified across various cell/tissue types, we constructed a positive set (RNA editing sites) and negative set (SNPs and other SNVs) using 64 RNA-seq samples of 32 cells/tissues (two replicates per cell line) from the ENCODE project^38^. For each cell type, we used STAR^46^ (Version: 2.5.2b) and GATK^43^ (version 3.5.0) to call SNVs from RNA-seq data, then used the separate samples or pooled samples method proposed by Li’s group to identify the RNA editing sites^27^ (see “Supplemental materials”). Thus, the candidate SNVs were classified into RNA editing sites, SNPs and other. Then, we constructed separate and pooled gold standard sets for each cell using the RNA editing sites identified by the separate samples and pooled samples methods as the positive set and using SNPs and other SNVs as the negative set (Table S1). The ratio of A-to-I editing sites in the positive and negative sets are 86.3% and 34.8%, respectively (Fig. S21). Of the 32 cells/tissues, we carefully selected 11 cells/tissues and used their separate and pooled gold standard sets to compose the separate and pooled training sets, respectively. Of the remaining 21 cells/tissues, we used their separate and pooled gold standard sets to make up the separate and pooled test sets, respectively (Fig. S1).

### Architecture and training of single-cell module

We designed the single-cell module to be a two-level ensemble classifier that used a simple averaging method to combine multiple ensemble Deep Neural Networks (DNNs) together (Fig. S3A). We adopted a bottom-up approach to train the single-cell module using the gold standard sets in Sknshra cells. The bagging-style bootstrapped resampling method was used to solve the class-imbalanced problem by separately sampling the same number of RNA editing sites, SNPs, and other classes (Fig. S4, see “Supplemental materials”).

First, we optimized the structure of the individual DNN in ensemble DNN. Each individual DNN took the 201-bp primitive sequence centred at candidate SNVs as an input. One-hot-encoding was applied to encode the bases “A”, “T”, “C”, and “G” of the 201-bp primitive sequence as (1, 0, 0, 0), (0, 1, 0, 0), (0, 0, 1, 0) and (0, 0, 0, 1), respectively. We fixed the number of hidden units in each hidden layer to 10, 50, 100, 500 and 1,000, and we tuned the structure of hidden layers using modified 5-fold cross-validation (See “Supplemental materials”) by grid search. In total, up to four hidden layers were searched. The optimized structure of individual DNNs consisted of two hidden layers with 1,000 units in the first hidden layer, 100 units in the second hidden layer, and a Softmax output layer with 3 output units.

Then, we used modified 5-fold cross-validation to assess the performance of ensemble DNN with different numbers of individual DNNs with the optimized structure (Fig. S2). The performance of the ensemble DNN was improved by increasing the number of individual DNNs; however, the AUC became almost saturated when the number reached 10. We set the number of individual DNNs to 20 to achieve a good trade-off between performance and computational resources. The AUC of the ensemble DNN in Sknshra cells is 0.970 (Fig. S2).

Next, we tried to determine the optimal number of ensemble DNNs for the single-cell module and find a better input scale of the primitive sequence surrounding candidate SNVs. We tried three other scales, namely, 101-bp, 41-bp and 21-bp primitive sequences centred at the candidate SNVs, and assessed the performance of 15 combinations of the four input scales with at most four ensemble DNNs (C_4_^1^ + C_4_^2^ + C_4_^3^ + C_4_^4^ = 15). We found that the 101-bp and 41-bp scale combination achieved the best performance. Thus, the single-cell module combined two ensemble DNNs consisting of 20 individual DNNs each with input scales of 101 bp and 41 bp (Figs S3B and S3C).

Finally, we evaluated the performance of the single-cell module on U87 data^33^ using modified 5-fold cross-validation (Fig. S5). The single-cell module of DeepRed achieved 0.933 AUC and 86.6% geometric mean (GM), suggesting that the single-cell module illustrates superior performance. Then, we used the optimized structure of the single-cell module as the basic unit of the separate and pooled components of DeepRed.

### Assessing hybrid structure of DeepRed

Our DeepRed method is a hybrid framework integrating deep learning and ensemble learning in which the input data flow from the lowest level individual DNN of the single-cell module to the highest level DeepRed, i.e., from the individual DNN, ensemble DNN, single-cell module, and separate/pooled component to DeepRed. We assessed the performance of the hybrid structure of DeepRed at each level using the training set and independent test set (Fig. S9). We found that the average AUC increased in a level-wise manner and that the deviation of AUC decreased in a level-wise manner. Our result demonstrated that the hybrid structure of DeepRed, which integrated deep learning with ensemble learning, promoted the identification accuracy of RNA editing, further confirming the advantages of the hybrid framework of DeepRed.

### Validation of DeepRed with experimentally verified data

We validated whether DeepRed identified bona fide RNA editing sites by applying it to experimentally verified data from U87, K562 and HepG2 cell lines using the method described in a previous study^27^ (Table S3). The validation flow in U87 is described in Fig. S10. We identified 26,985 and 6,777 candidate RNA editing sites using the experimental samples from wild-type and knock-down U87, respectively. When we compared the difference in RNA editing sites between wild-type and knock-down U87 samples, we obtained 26,059 true and 6,777 false RNA editing sites from experimentally verified data (Fig. S11A). Then, we called 12,609 candidate SNVs using the independent U87 test sample from ENCODE. Of these 12,609 candidate SNVs, 192 sites were experimentally verified by comparing the RNA editing sites from the experimental and test samples, including 59 true RNA editing sites and 133 false RNA editing sites. Next, we applied DeepRed to identify RNA editing from the 12,609 candidate SNVs of the U87 test sample. We evaluated the performance of DeepRed based on the 192 experimentally verified editing sites (Fig. S11B) using eight performance indicators, including accuracy; sensitivity; specificity; GM; positive predict value; F1-score; validation rate; and misclassification rate (Table 1). Furthermore, we applied the separate samples, GIREMI, RNAEditor and Prediction method to the U87 test sample and performed the same validation analysis. The compared performance of these five methods is summarized in Table 1. The same validation and comparison analysis was also performed on K562 and HepG2 cell lines (Tables S5 and S6).

### Performance comparison of DeepRed with existing methods

We applied DeepRed, separate samples, pooled samples, and GIREMI methods to the 462 RNA-seq datasets of lymphoblastoid cells from the Geuvadis Project^37^ to compare the performance of our DeepRed method to three other state-of-the-art methods (Table S7). We compiled all RNA editing sites identified from 462 individuals for each method and compared the performance of these methods based on the number of RNA editing sites, A-to-I ratio and false discovery rate (FDR). In addition, we compared the performance of these methods in each individual and plotted the distribution of the A-to-I ratio and FDR of each method. We called the variance of the A-to-I ratio in 462 individuals, then compared the consistency of these methods. Furthermore, we performed the same comparison analysis on RNA-seq samples from ENCODE^38^, Roadmap Epigenomics^39^, and cancer cell line encyclopedia (CCLE)^40^ projects (Tables S15–S17).

We down sampled the RNA-seq data of human brain reference data from the SEQC project to compare the overall runtime of DeepRed with the separate samples and GIREMI methods (Table S9). Since the runtimes of sequence read mapping and SNV calling were equal for these three methods, we compared only the runtimes of calling RNA editing sites from the SNVs. We calculated the runtime across ten down sampling samples at each sequence depth.

### Assessing impact of multiple factors on RNA editing identification

We examined a series of factors and their impact on identification of RNA editing, including library preparation, RNA degradation, laboratory, sequence depth, read mapping and variant calling, by applying DeepRed to RNA-seq data from the ABRF^35^ and SEQC^36^ projects (Tables S9–S11, see “Supplemental materials”). We used the identified number, recognition accuracy and reproducibility of RNA editing sites to assess the impact of different conditions on RNA editing identification. Recognition accuracy refers to the percentage of A-to-I editing sites. Reproducibility is the overlap ratio of RNA editing sites identified in two different conditions.

### Application analysis of DeepRed across species

We applied our DeepRed method to identify RNA editing sites in the oocyte, zygote, 2-cell, 4-cell, 8-cell, morula, and late blastocyst stages^47^ to explore the developmental pattern of RNA editing during human early embryogenesis (Table S12). We down sampled 10 million mapped reads per cell at each stage to remove sequence depth bias from the number of identified RNA editing sites. We examined the evolutionary conservation of RNA editing across species by first identifying RNA editing sites in *Drosophila* species (*D. melanogaster*, *D. simulans*, *D. ananassae*, *D. pseudoobscura*, *D. mojavensis*, and *D. virilis*)^48^ (Table S13) and the primate lineage (human, chimpanzee, rhesus macaque and mouse)^49^ (Table S14). For human and mouse analyses, SNVs were called using GATK^43^ (Version: 3.5.0), whereas for *Drosophila* analyses, SNVs were called using SAMtools^54^ (Version: 1.3.1) (see “Supplemental materials”). Information on the reference genome, dbSNPs and gene model used in this study are listed in Table S18. The LiftOver tool was used to convert genomic positions between different species. Homologous RNA editing sites are RNA editing sites of one species that have orthologous sites in another species. The homologous ratio is the number of homologous RNA editing sites compared to the number of identified RNA editing sites.

### Availability

The source code for our DeepRed method can be freely accessed at https://github.com/wenjiegroup/DeepRed.

## Electronic supplementary material

Supplementary info

Table S1

Table S2

Table S3

Table S4

Table S5

Table S6

Table S7

Table S8

Table S9

Table S10

Table S11

Table S12

Table S13

Table S14

Table S15

Table S16

Table S17

Table S18
