# Supplementary material for: Accurate identification of RNA editing sites from primitive sequence with deep neural networks
Source: Sci Rep. 2018 Apr 16;8:6005. doi: 10.1038/s41598-018-24298-y (PMC5902551; doi:10.1038/s41598-018-24298-y)
Supplement: Supplementary file 1 — Supplementary info [file 41598_2018_24298_MOESM1_ESM.pdf]

Supplementary material for

Accurate identification of RNA editing sites from primitive sequence with deep neural networks

Zhangyi Ouyang[1, †], Feng Liu[2, †], Chenghui Zhao[1], Chao Ren[1], Gaole An[1], Chuan Mei [3], Xiaochen Bo[1,*], Wenjie Shu[1,*]

[1] Department of Biotechnology, Beijing Institute of Radiation Medicine, Beijing, 100850, China

[2] Department of information, the 188th hospital of ChaoZhou, ChaoZhou, 521000, China

[3] Department of medical services, the 188th hospital of ChaoZhou, ChaoZhou, 521000, China

* To whom correspondence should be addressed. Tel: +86 10 66932211; Fax: +86 10 68210077; Email: shuwj@bmi.ac.cn. Correspondence may also be addressed to: boxc@bmi.ac.cn.

[†] These authors contributed equally to this work.

SUPPLEMENTAL METHODS

Modified 5-fold cross-validation

Traditional 5-fold cross-validation involves partitioning a sample of data into five complementary subsets, performing analysis on the four subsets (called the training set), and validating the analysis on the other subset (called the test set). Considering the class-imbalance data in this study, the test set is likely to contain only negative samples and no positive samples, which thus affects the assessment of model performance. Therefore, we used modified 5-fold cross-validation to evaluate performance. First, we randomly divided the positive and negative sets into five complementary and equally sized subsets. Then, we selected four positive and four negative subsets as the training set. In addition, the remaining one positive subset and one negative subset comprised an independent test set. The process was then repeated 5 times with each of the 5 positive subsets and negative subsets used exactly once as a test set.

Bagging-style method solved the class-imbalanced problem

We introduced three popular parallel ensemble methods that were based on deep learning, including classic bagging, modified bagging, and bagging-style bootstrapped resampling methods [1], to develop a flexible single-cell module of DeepRed that could dynamically solve the class-imbalanced problem and be easily expanded to various cells/tissues and even other species. Although partition of the majority class method is a typical approach for class-imbalanced data, it is a static method and does not satisfy our demand that the imbalance degree can dynamically change. Thus, we excluded this method to solve the class-imbalanced problem. To select the most suitable method from the three bagging-style methods for DeepRed, we tested and evaluated them independently using class-imbalanced data of various imbalance degrees (ratio of negative samples/positive samples) in two cell types (Fig. S4). For a fair comparison, the number and structure of individual DNN classifiers were kept the same (10 individual DNNs with the same structure). Three performance indicators, namely, sensitivity, specificity and GM, were reported as suitable for assessing imbalanced data sets and were measured using modified 5-fold cross-validation. We observed that for both the classic bagging and modified bagging methods, sensitivity and GM decreased rapidly and specificity increased slightly with an increasing imbalance degree of class-imbalanced data (Fig. S4A-D). The result suggested that these two methods tend to be biased towards the majority class and cannot alleviate the class-imbalanced problem. In contrast, for the bagging-style bootstrapped resampling method, the three performance indicators were all unbiased for different imbalance degrees of class-imbalanced data (Fig. S4E-F), suggesting that the bagging-style bootstrapped resampling method can sufficiently eliminate the class-imbalanced influence. Thus, we used the bagging-style bootstrapped resampling method in the single-cell module of DeepRed to process dynamic imbalance-class data.

Simple average as combination method of ensemble learning

As for the combination methods of ensemble learning, we tried and evaluated three different methods, including simple averaging, weighted averaging, and stacking. In the simple averaging method, the predicted probabilities from each individual DNNs were averaged to produce a single estimation. We set the weight of the weighted average according to the AUC or accuracy of the single-cell module and found that the difference between the weighted

average and simple average was negligible. Although the stacking method improved model performance to some extent, the boost in performance was extremely limited (less than 1% for AUC). The weighted averaging and stacking methods achieved 0.0045% and 0.7482% higher AUC than the simple averaging method, respectively. In addition, stacking integration doubled the computing time and computing resources and had the limited scalability. Importantly, simple averaging can be extended to additional classifiers based on the original training; however, stacking must be re-trained for the integration of an additional classifier. Considering computing time, computing resources, and more importantly, expandability, we chose simple averaging as the final combination method for ensemble learning.

SNV calling method

The reference genome, dbSNP and gene model used in this study are listed in Table S18. We used STAR [2] (Version: 2.5.2b) to align RNA-seq reads to a reference genome and used the MarkDuplicates tool from Picard (https://broadinstitute.github.io/picard/) to remove identical reads (PCR duplicates) that mapped to the same location. Reads with a mapping quality < 20 were removed by SAMtools (Version: 1.3.1) [3]. For human and mouse, SNVs were called using the GATK [4] (Version: 3.5.0) HaplotypeCaller tool with options stand_call_conf set at 20 and stand_emit_conf set at 0. For *Drosophila*, SNVs were called using the SAMtools pileup program with option "-Q 15" due to the insufficiency of SNP information in *Drosophila* species.

Identifying RNA editing sites with existing methods

In the separate samples method, SNVs are called with separate RNA-seq alignments of each sample, and reoccurring variants are retrieved [5]. In the pooled samples method, SNVs are called with pooled RNA-seq alignments from all samples [5]. The SNVs were filtered by five steps: 1) all known SNPs present in dbSNP (except SNPs of molecular type "cDNA") were removed; 2) mismatches in the first six bases of each read were discarded to avoid artificial mismatches derived from random-hexamer priming; 3) intronic sites were removed in non-*Alu* regions if they were located within 4 base pairs of a known splice junction, and sites in homopolymer runs of 5 base pairs and simple repeats were removed; 4) sites in regions that were highly similar to

other parts of the genome using the BLAST-like alignment tool (BLAT) were removed; (5) We inferred the editing type of each site based on the strand of overlapping annotated genes.

In GIREMI method [6], SNVs were filtered by five steps: 1) mismatches in the first six bases of each read were discarded to avoid artificial mismatches derived from random-hexamer priming; 2) sites located in simple repeat regions or homopolymer runs of 5 nt were discarded; 3) variant sites with total read coverage < 5 and supporting reads < 3 were discarded; 4) sites with extreme variant allele frequencies (>95% or <10%) were discarded; and 5) sites located within 4 nt of a known spliced junction were removed. The remaining SNVs were annotated in the required format for GIREMI using GENCODE (V25 lift37). Then, we ran GIREMI to calculate mutual information (MI) associated with SNPs or RNA editing sites and identify RNA editing sites in RNA-seq reads. GIREMI was downloaded from https://github.com/zhqingit/giremi.

In Prediction method [7], SNVs were retained as RNA editing to meet these five criterias: 1) mismatch sites with Hits Per Billionmapped-bases (HPB) > 5; 2) mismatch sites with mismatch ratio between 5% ~ 40% or between 60% ~ 95%; 3) mismatch sites with effective signal > 95%; 4) require at least two individual reads with the same type of nucleotide conversion; 5) mismatch sites do not exist in gSNPs from the common SNP database (build 137).

RANEditor is an easy-to-use tool [8]. We used the script CallingEditingSites.py from RNAEditor package to call RNA editing sites.

Assessing multiple factors on identifying RNA editing sites

We examined a series of factors, including library preparation methods, RNA degradation methods, laboratory, sequence depth, read mapping and variant calling methods, and their impact on the identification of RNA editing by applying DeepRed to RNA-seq data from the ABRF [9] and SEQC project [10].

We assessed the impact of library preparation methods and RNA degradation methods by analysing 34 samples from the ABRF project [9] (Table S10), 8 of which were intact RNA prepared by the poly-A enrichment method, 8 that were intact RNA prepared by the ribo-depleted method, and 18 that were degraded RNA prepared by the ribo-depleted method. The

degraded samples were degraded using one of three methods, namely, heat, sonication or RNase-A.

Widespread adoption of RNA-seq has led to plentiful data from multiple sites; however, there has been no systematic examination of the impact of lab-specific bias in detecting RNA editing sites. We explored the effect of laboratory by analysing 94 samples collected from 7 laboratories, including AGR (Australian Genome Research Facility), BGI (Beijing Genomics Institute), CNL (Weill Cornell Medical College), COH (City of Hope), MAY (Mayo Clinic), NVS (Novartis), and NYG (the New York Genome Center) (Table S11).

We pooled together human brain RNA-seq samples of 16 individuals from the SEQC project [10] (Table S9) and down sampled the pooled alignment file to explore the relationship between the detection of RNA editing sites and sequence depth to examine the impact of the sequence depth. For each sequence depth, we sampled the corresponding RNA-seq reads and calculated the A-to-I ratio and number of A-to-I editing sites. We repeated this down sampled analysis 10 times for each sequence depth, then calculated the average values.

RNA-seq read mapping and variant calling is an important step in the detection of RNA editing sites. Various read mapping and variant calling strategies can be adopted, such as BWA [11], STAR [2], Tophat [12], GATK [4] and SAMtools [3], but the best practice for detecting RNA editing sites has not been fully defined. To this end, we used the same pooled human brain RNA-seq alignment (Table S9) to perform read mapping and variant calling method comparisons. Different combinations of read mapping methods (BWA, STAR, and Tophat) and variant calling methods (GATK and SAMtools) were used to call SNV candidates. Then, DeepRed was employed to identify RNA editing sites in the SNV candidates. We compared the identified number of RNA editing sites, recognition accuracy and reproducibility to assess the impact of different conditions on RNA editing identification. Recognition accuracy refers to the percentage of A-to-I editing sites. Reproducibility is the overlap ratio of RNA editing sites identified in two different conditions.

REFERENCES

1 He, H. & Ma, Y. Imbalanced learning. *Wiley & Sons* (2013).

2 Dobin, A. *et al.* STAR: ultrafast universal RNA-seq aligner. *Bioinformatics* **29**, 15-21, doi:10.1093/bioinformatics/bts635 (2013).
3 Li, H. *et al.* The Sequence Alignment/Map format and SAMtools. *Bioinformatics* **25**, 2078-2079, doi:10.1093/bioinformatics/btp352 (2009).
4 DePristo, M. A. *et al.* A framework for variation discovery and genotyping using next-generation DNA sequencing data. *Nat Genet* **43**, 491-498, doi:10.1038/ng.806 (2011).
5 Ramaswami, G. *et al.* Identifying RNA editing sites using RNA sequencing data alone. *Nat Methods* **10**, 128-132, doi:10.1038/nmeth.2330 (2013).
6 Zhang, Q. & Xiao, X. Genome sequence–independent identification of RNA editing sites. *Nat Methods* **12**, 347-350, doi:10.1038/nmeth.3314 (2015).
7 Zhu, S., Xiang, J. F., Chen, T., Chen, L. L. & Yang, L. Prediction of constitutive A-to-I editing sites from human transcriptomes in the absence of genomic sequences. *BMC Genomics* **14**, 206, doi:10.1186/1471-2164-14-206 (2013).
8 John, D., Weirick, T., Dimmeler, S. & Uchida, S. RNAEditor: easy detection of RNA editing events and the introduction of editing islands. *Brief Bioinform* **18**, 993-1001, doi:10.1093/bib/bbw087 (2017).
9 Li, S. *et al.* Multi-platform assessment of transcriptome profiling using RNA-seq in the ABRF next-generation sequencing study. *Nat Biotechnol* **32**, 915-925, doi:10.1038/nbt.2972 (2014).
10 Consortium, S. M.-I. A comprehensive assessment of RNA-seq accuracy, reproducibility and information content by the Sequencing Quality Control Consortium. *Nat Biotechnol* **32**, 903-914, doi:10.1038/nbt.2957 (2014).
11 Li, H. & Durbin, R. Fast and accurate short read alignment with Burrows-Wheeler transform. *Bioinformatics* **25**, 1754-1760, doi:10.1093/bioinformatics/btp324 (2009).
12 Trapnell, C., Pachter, L. & Salzberg, S. L. TopHat: discovering splice junctions with RNA-Seq. *Bioinformatics* **25**, 1105-1111, doi:10.1093/bioinformatics/btp120 (2009).

SUPPLEMENTARY TABLE LEGENDS

Supplementary Table S1. The information of 11 training and 21 test cells cell lines/tissues/conditions from Encode.

Supplementary Table S2. Performance of each-level DNNs in DeepRed on training set and test set.

Supplementary Table S3. The information of wild type, ADAR knock down and independent test data for validation of DeepRed.

Supplementary Table S4. Relative ranking of DeepRed, separate samples method, GIREMI, RNAEditor and Prediction methods on U87 data.

Supplementary Table S5. Comparison of performance for DeepRed with separate samples method, GIREMI, RNAEditor and Prediction methods in K562 cell line.

Supplementary Table S6. Comparison of performance for DeepRed with separate samples method, GIREMI, RNAEditor and Prediction methods in HepG2 cell line.

Supplementary Table S7. Information of 462 individuals from the Geuvadis Project.

Supplementary Table S8. Performance of DeepRed in comparison with Jin Billy methods and GIREMI on RNA-seq data in Geuvadis project (didn't filter reads).

Supplementary Table S9. RNA-seq data of human brain reference from SEQC project.

Supplementary Table S10. RNA-seq data prepared by different library methods and degraded by different methods from ABRF project.

Supplementary Table S11. RNA-seq data sequenced by different laboratories in SEQC project.

Supplementary Table S12. RNA-seq data from human embryos spanning from oocyte to late blastocyst stages.

Supplementary Table S13. RNA-seq data from adult whole bodies of D. melanogaster, D. simulans, D. ananassae, D. pseudoobscura, D. mojavensis, and D. virilis.

Supplementary Table S14. RNA-seq data of human, chimpanzee, rhesus macaque and mouse brain.

Supplementary Table S15. The information of samples from ENCODE project.

Supplementary Table S16. The information of samples from Roadmap Epigenomics project.

Supplementary Table S17. The information of samples from CCLE project.

Supplementary Table S18. Information of reference, dbsnp, gene model used in this study.

SUPPLEMENTARY FIGURES LEGENDS

Supplementary Figure S1. The construction of training and test sets. (A) The construction of training set. (B) The construction of test set.

Supplementary Figure S2. Performance of ensemble DNN under different numbers of individual DNNs in Sknshra cell.

Supplementary Figure S3. The two-level ensemble architecture of single-cell module. (A) The single-cell module is a two-level ensemble classifier, which combined two ensemble Deep Neural Networks (DNNs) (light blue and light purple) with input scale of 101 bp and 41bp primitive sequence centered at the candidate SNVs. Each ensemble DNN consisted of 20 individual DNNs, and was combined together using simple averaging method. For each individual DNN, the sigmoid function is used as the active function. (B) In one ensemble DNNs (light blue), each individual DNN consisted of one input layer with 404 input units, two hidden layers with the first hidden layer containing 1000 hidden units and the second hidden layer containing 100 hidden units, and a Softmax output layer with 3 output units. (C) In another ensemble DNNs (light purple), each individual DNN consisted of one input layer with 164 input units, two hidden layers with the first hidden layer containing 1000 hidden units and the second hidden layer containing 100 hidden units and a Softmax output layer with 3 output units.

Supplementary Figure S4. Performance of three ensemble methods in class-imbalanced data. The performance of three deep-learning-based ensemble methods, including classic bagging (AB), modified bagging (CD) and bagging-style bootstrapped resampling method (EF), in class-imbalanced data of various degrees in Nhek cells (ACE) and Sknshra Cells (BDF). Three performance indicators, sensitivity, specifcity, and GM, were measured for the training set.

Supplementary Figure S5. ROC of single-cell module for DeepRed in U87 cell.

Supplementary Figure S6. The separate and pooled components of DeepRed. (A) The separate or pooled component of DeepRed combined 11 single-cell modules using simple averaging method. (B) DeepRed combined separate and pooled component using simple averaging method.

Supplementary Figure S7. The pseudocode shows the detailed training steps of DeepRed.

Supplementary Figure S8. Precision recall curve of DeepRed on training sets (A) and test sets (B).

Supplementary Figure S9. Assessing hybrid structure of DeepRed. Performance assessment of each-level DNNs in DeepRed on 11 pooled training sets (A), 11 separate training sets (B), 21 pooled test sets (C) and 21 separate test sets (D). The violin plot represents the distribution of AUC of individual DNN. Red cross indicates the average performance.

Supplementary Figure S10. The validation of DeepRed on U87 experimentally-verified data.

Supplementary Figure S11. The RNA editing sites used to validate the effectiveness of DeepRed. (A) The identification of true RNA editing sites and false RNA editing sites from wild-type and knock-down sample. (B) The RNA editing sites used to validate the effectiveness of DeepRed.

Supplementary Figure S12. Performance of DeepRed in comparison with separate samples method and GIREMI method. (A) The violin plot of FDR identified by DeepRed, separate samples method and GIREMI method for each individual in Geuvadis dataset. The first, second (median), and third quartiles were illustrated in a box-plot style. (B) The relationship between the sequence depth and the runtime of the RNA editing identified with DeepRed, separate samples method and GIREMI method. The insert plot represents the relationship between sequence depth and runtime of RNA editing identification in DeepRed. The runtime refers to the time spend on identifying RNA editing sites from candidates SNVs. Error bar represents the standard error of runtime across ten downsampling samples.

Supplementary Figure S13. The A-to-I ratio and FDR of RNA editing sites identified by DeepRed, separate samples method and GIREMI method for each individual in Encode (A-B), roadmap (C-D), and CCLE (E-F) dataset. The first, second (median), and third quartiles were illustrated in a box-plot style.

Supplementary Figure S14. The site importance score of bases flanking RNA editing sites for prediction.

Supplementary Figure S15. The A-to-I ratio, FDR and reproducibility of RNA editing sites between RNA-seq prepared by different library methods and degraded by different methods.

The A-to-I ratio, FDR (A), and reproducibility (B) of RNA editing sites in RNA-seq prepared by different library methods and degraded by different methods.

Supplementary Figure S16. Assessment of impact of different laboratories on RNA editing identification. The number of identified RNA editing sites in each laboratory. A-to-I ratio and FDR of identified RNA editing sites in each laboratory.

Supplementary Figure S17. The A-to-I ratio and number of A-to-I editing sites identified by separate samples method at different sequence depth.

Supplementary Figure S18. Assessment of impact of different read mapping and variant calling methods on RNA editing identification. (A) The A-to-I ratio and FDR for different combination of read mapping method and variant calling method. (B) The number of A-to-I editing sites for different combination of read mapping method and variant calling method.

Supplementary Figure S19. The number of RNA editing sites identified by separate sample method in human early embryogenesis.

Supplementary Figure S20. The conservation analysis between primate lineages. Phylogenetic relationships among the primate lineage (left). Myr, million years ago. Ratio of RNA editing sites homologous to human in primate lineage (right).

Supplementary Figure S21. The distribution of 12 possible mismatches in positive training set and negative training set.

A

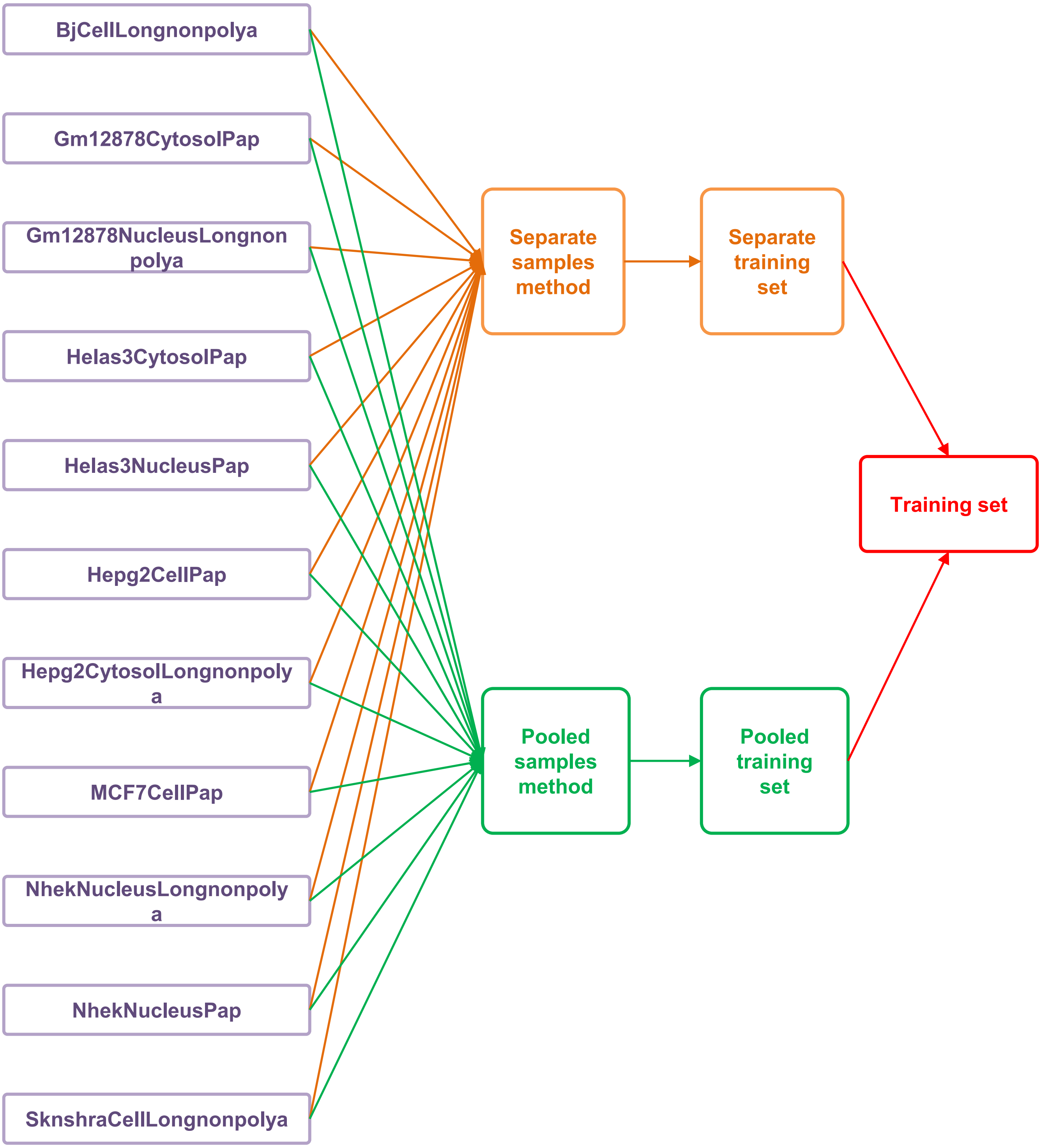

BjCellLongnonpolya
Gm12878CytosolPap
Gm12878NucleusLongnonpolya
Helas3CytosolPap
Helas3NucleusPap
Hepg2CellPap
Hepg2CytosolLongnonpolya
MCF7CellPap
NhekNucleusLongnonpolya
NhekNucleusPap
SknshraCellLongnonpolya
Separate samples method
Separate training set
Pooled samples method
Pooled training set
Training set

Fig. S1

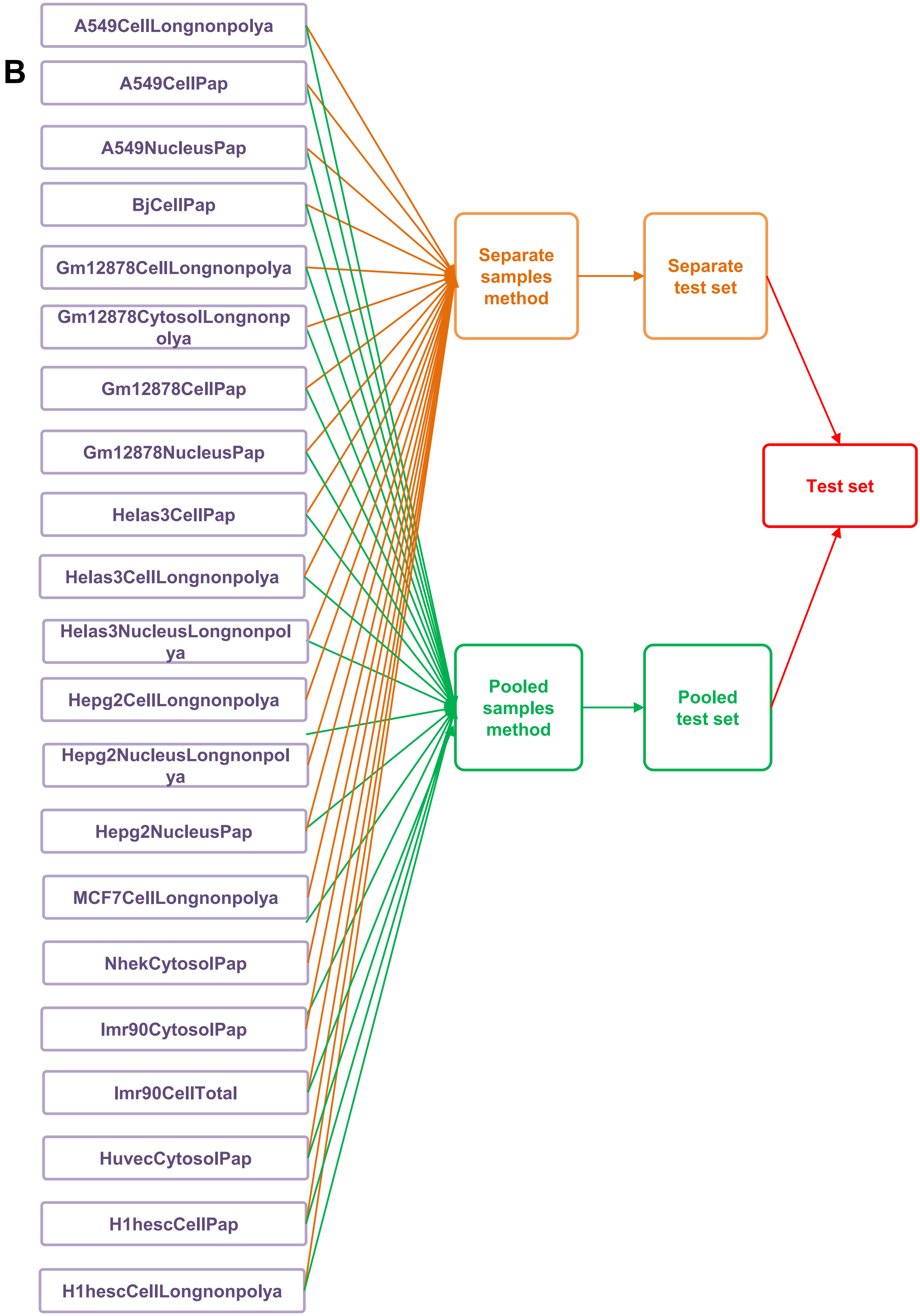
B
A549CellLongnonpolya
A549CellPap
A549NucleusPap
BjCellPap
Gm12878CellLongnonpolya
Gm12878CytosolLongnonpolya
Gm12878CellPap
Gm12878NucleusPap
Helas3CellPap
Helas3CellLongnonpolya
Helas3NucleusLongnonpolya
Hepg2CellLongnonpolya
Hepg2NucleusLongnonpolya
Hepg2NucleusPap
MCF7CellLongnonpolya
NhekCytosolPap
Imr90CytosolPap
Imr90CellTotal
HuvecCytosolPap
H1hescCellPap
H1hescCellLongnonpolya
Separate samples method
Separate test set
Test set
Pooled samples method
Pooled test set

Fig. S1

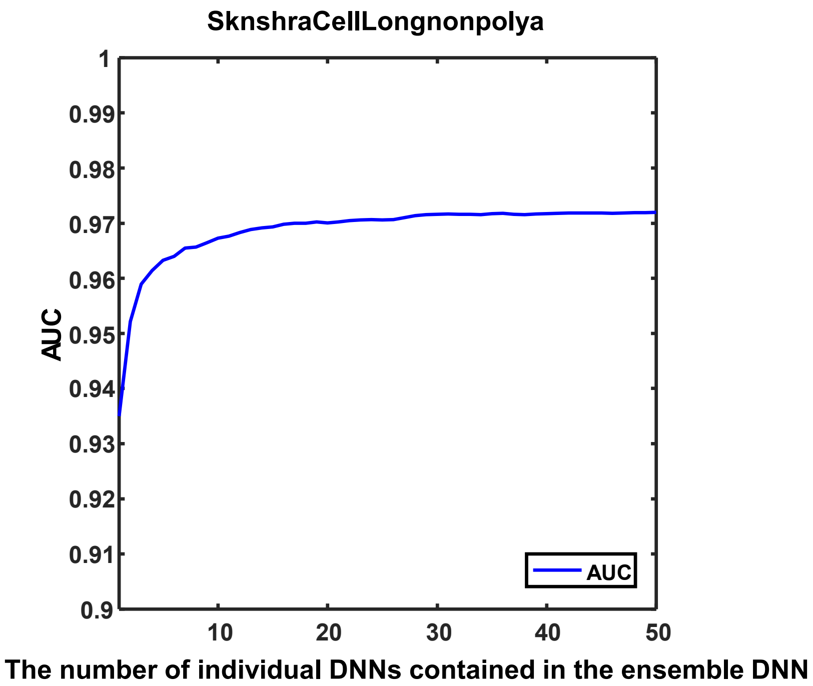
SknshraCellLongnonpolya
AUC
1
0.99
0.98
0.97
0.96
0.95
0.94
0.93
0.92
0.91
0.9
10
20
30
40
50
The number of individual DNNs contained in the ensemble DNN
AUC

Fig. S2

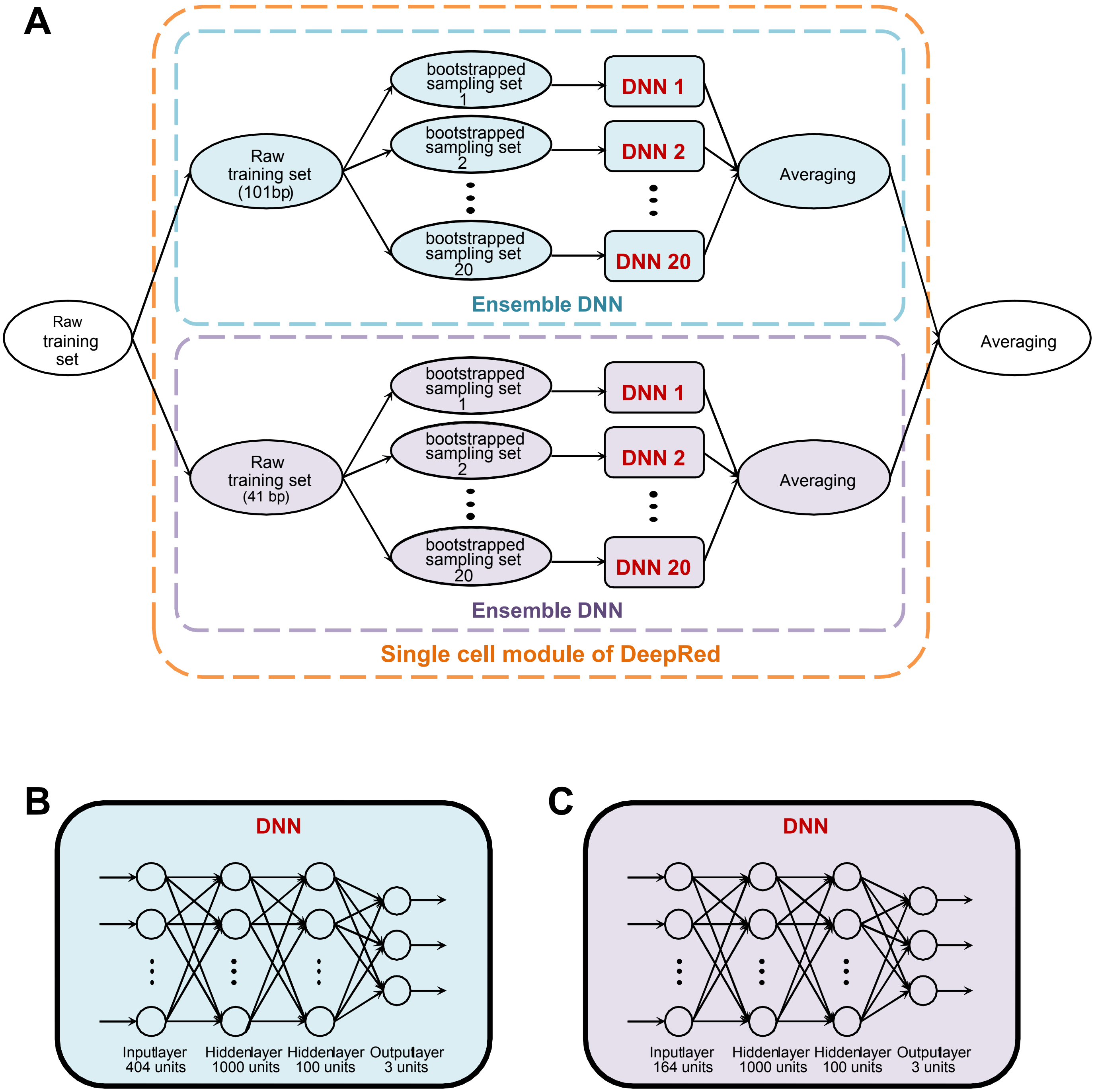
A
Raw training set
Raw training set (101bp)
bootstrapped sampling set 1
bootstrapped sampling set 2
bootstrapped sampling set 20
DNN 1
DNN 2
DNN 20
Averaging
Ensemble DNN
Raw training set (41 bp)
bootstrapped sampling set 1
bootstrapped sampling set 2
bootstrapped sampling set 20
DNN 1
DNN 2
DNN 20
Averaging
Ensemble DNN
Single cell module of DeepRed
Averaging
B
DNN
Inputlayer 404 units
Hiddenlayer 1000 units
Hiddenlayer 100 units
Outputlayer 3 units
C
DNN
Inputlayer 164 units
Hiddenlayer 1000 units
Hiddenlayer 100 units
Outputlayer 3 units

Fig. S3

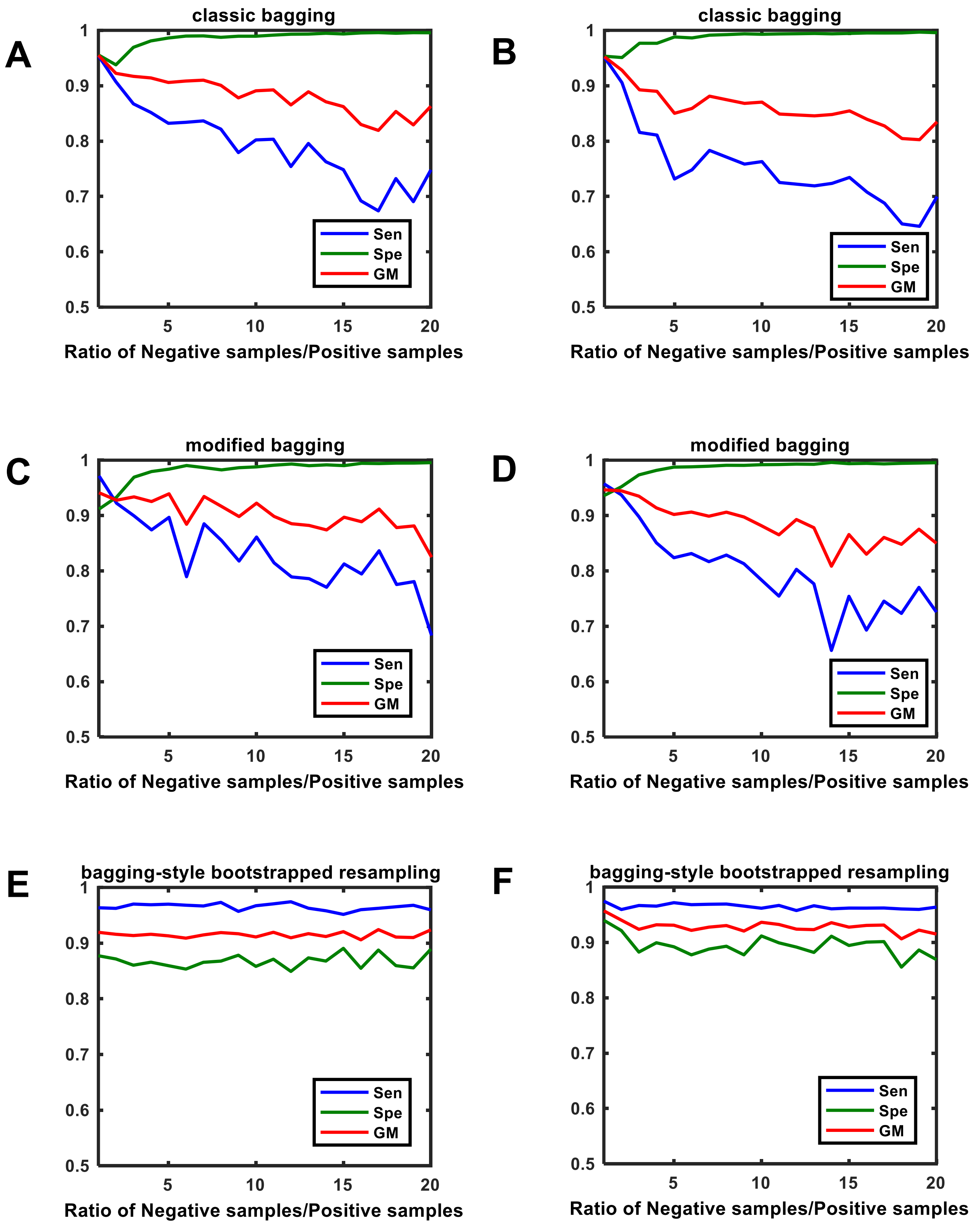

A
classic bagging
B
classic bagging
C
modified bagging
D
modified bagging
E
bagging-style bootstrapped resampling
F
bagging-style bootstrapped resampling
Sen
Spe
GM
1
0.9
0.8
0.7
0.6
0.5
5
10
15
20
Ratio of Negative samples/Positive samples

Fig. S4

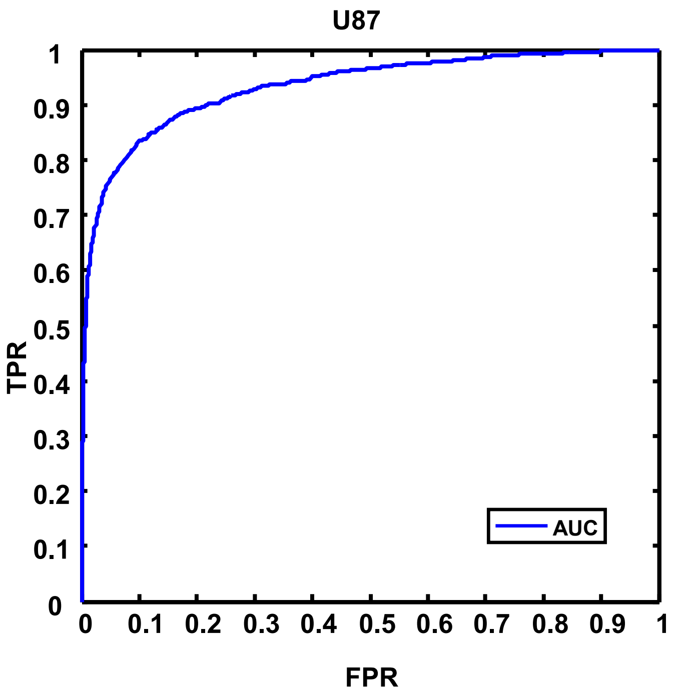
U87
TPR
FPR
AUC
1
0.9
0.8
0.7
0.6
0.5
0.4
0.3
0.2
0.1
0
0 0.1 0.2 0.3 0.4 0.5 0.6 0.7 0.8 0.9 1

Fig. S5

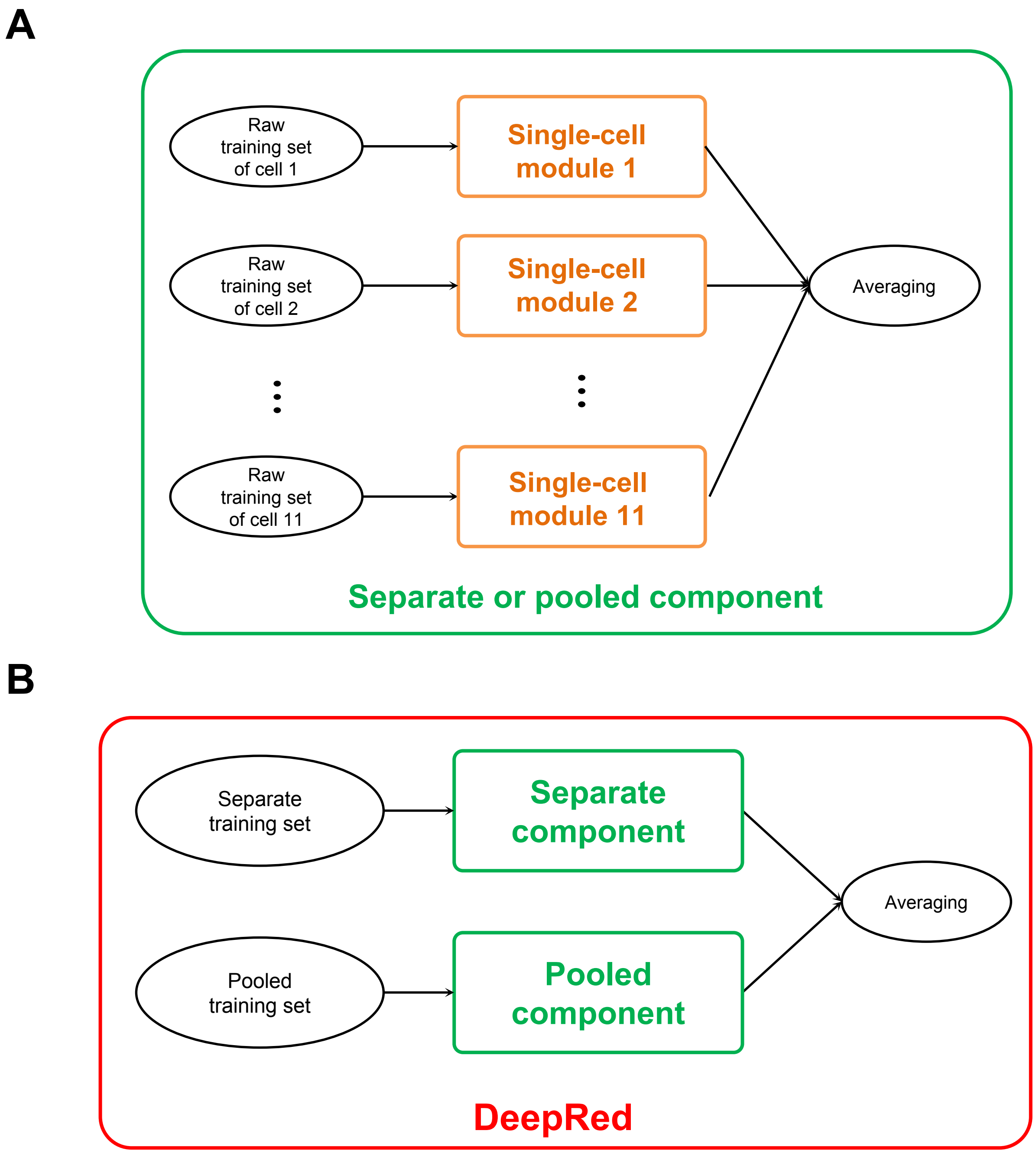
A
Raw training set of cell 1
Single-cell module 1
Raw training set of cell 2
Single-cell module 2
Raw training set of cell 11
Single-cell module 11
Averaging
Separate or pooled component
B
Separate training set
Separate component
Pooled training set
Pooled component
Averaging
DeepRed

Fig. S6

1. Training procedure of DeepRed:

Input: one-hot-encoded sequences and labels of training set
Output: the probability of being each class

for each component **do**

for each single-cell module **do**
for each ensemble DNN **do**
for each individual DNN **do**

1) resample class-balanced data with replacement from the raw class-imbalanced training set.
2) pre-train the DNN with sigmoid activation function layer by layer in an unsupervised fashion, then use the pre-training as initialization of neural network weights **W**. The sigmoid activation function is: $S(\mathrm{x}) = \frac{1}{1+e^{-x}}$
3) fine-tune the weights **W** of the DNN in term of cross-entropy using mini-batch gradient descent in a supervised manner. The cost function is:

$$\mathrm{J}(\theta) = -\frac{1}{m} \sum_{i=1}^{m} \sum_{j=1}^{k} 1\{y^{(i)} = j\} log \frac{e^{\theta_j^T x^{(i)}}}{\sum_{l=1}^{k} e^{\theta_l^T x^{(i}}} + \frac{\lambda}{2} \sum_{i=1}^{k} \sum_{j=0}^{n} \theta_{ij}^2$$

where k is the number of classes, m is the number of input samples and n is the dimension of feature.

end
end
end
end

1.

2. Prediction procedure of DeepRed:

Input: one-hot-encoded sequences of candidate SNVs
Output: the probability of being each class

for each component **do**

for each single-cell module **do**
for each ensemble DNN **do**
for each individual DNN **do**
forward propagate to get the posterior probability of each class based on the already-learnt weights **W**
end
average all the posterior probabilities of individual DNNs in the ensemble DNN
end
average all the posterior probabilities of ensemble DNN in the single-cell module
end
average all the posterior probabilities of single-cell modules in the component
end
average all the posterior probabilities of components as the final output of DeeRed.

Fig. S7

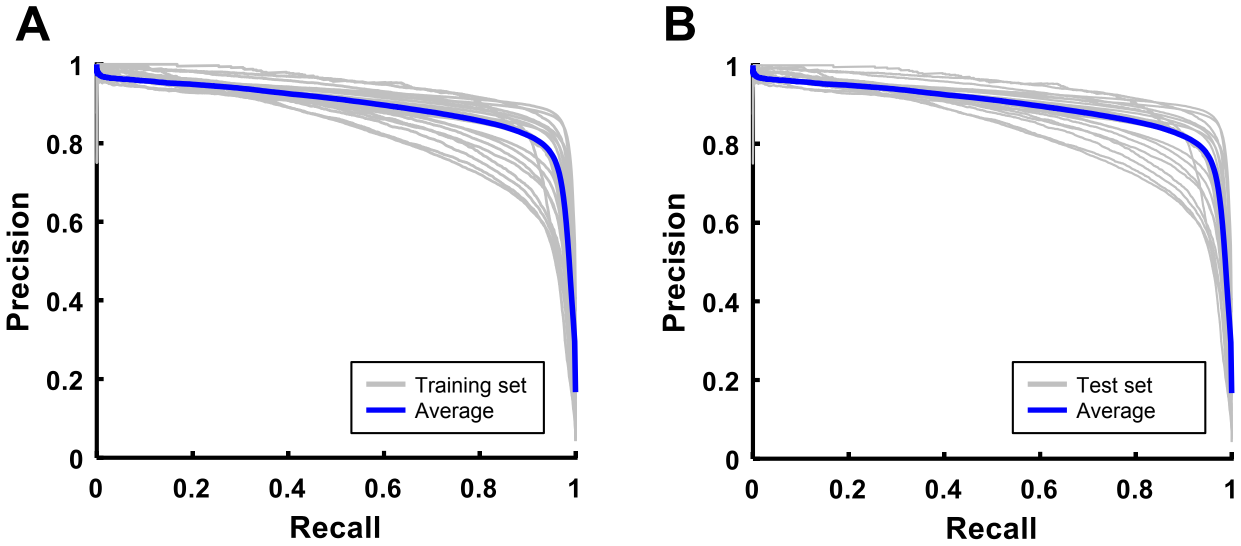

A
B
Precision
Recall
0
0.2
0.4
0.6
0.8
1
Training set
Test set
Average

Fig. S8

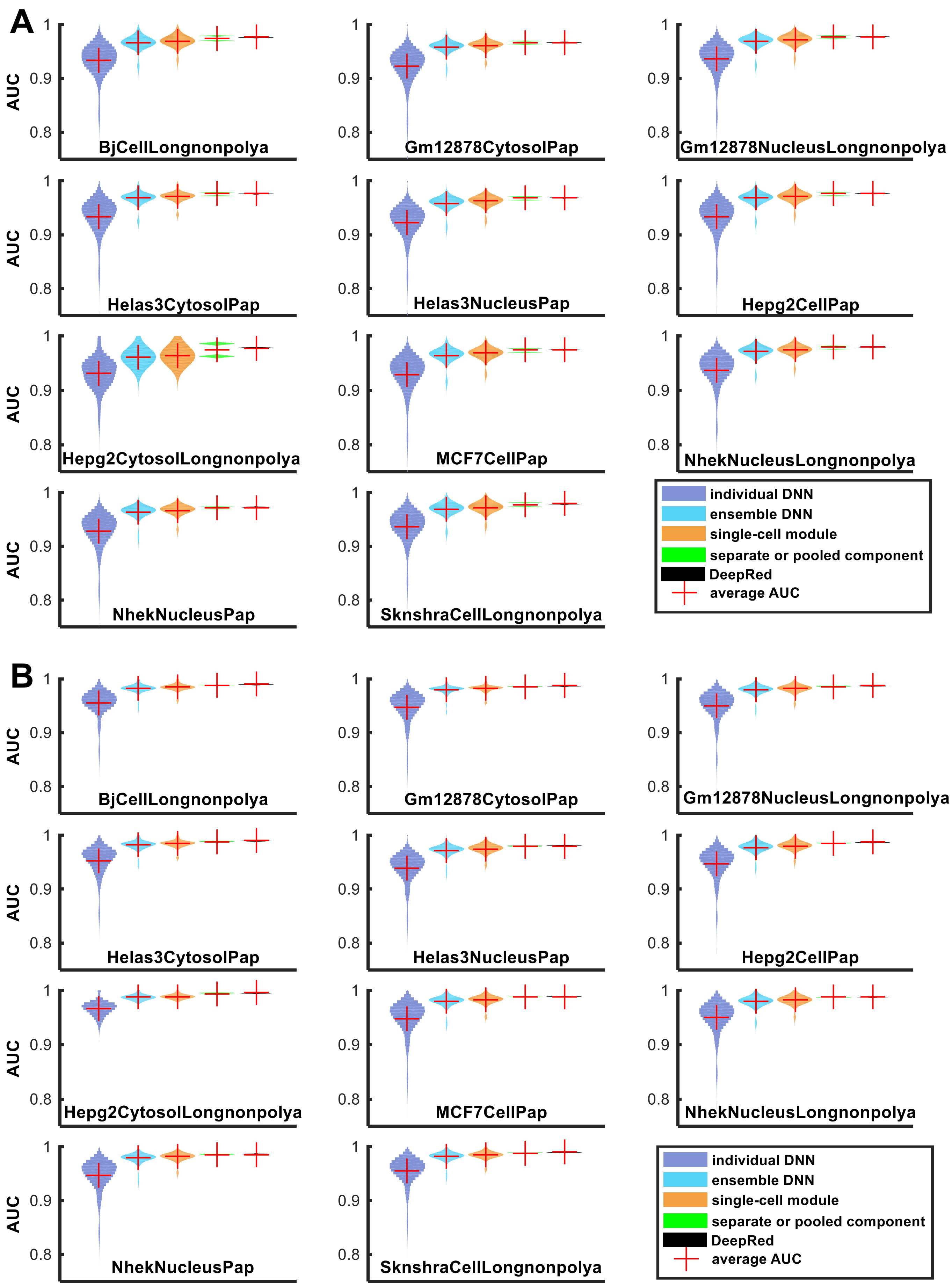
A
B
AUC
1
0.9
0.8
BjCellLongnonpolya
Gm12878CytosolPap
Gm12878NucleusLongnonpolya
Helas3CytosolPap
Helas3NucleusPap
Hepg2CellPap
Hepg2CytosolLongnonpolya
MCF7CellPap
NhekNucleusLongnonpolya
NhekNucleusPap
SknshraCellLongnonpolya
individual DNN
ensemble DNN
single-cell module
separate or pooled component
DeepRed
average AUC

Fig. S9

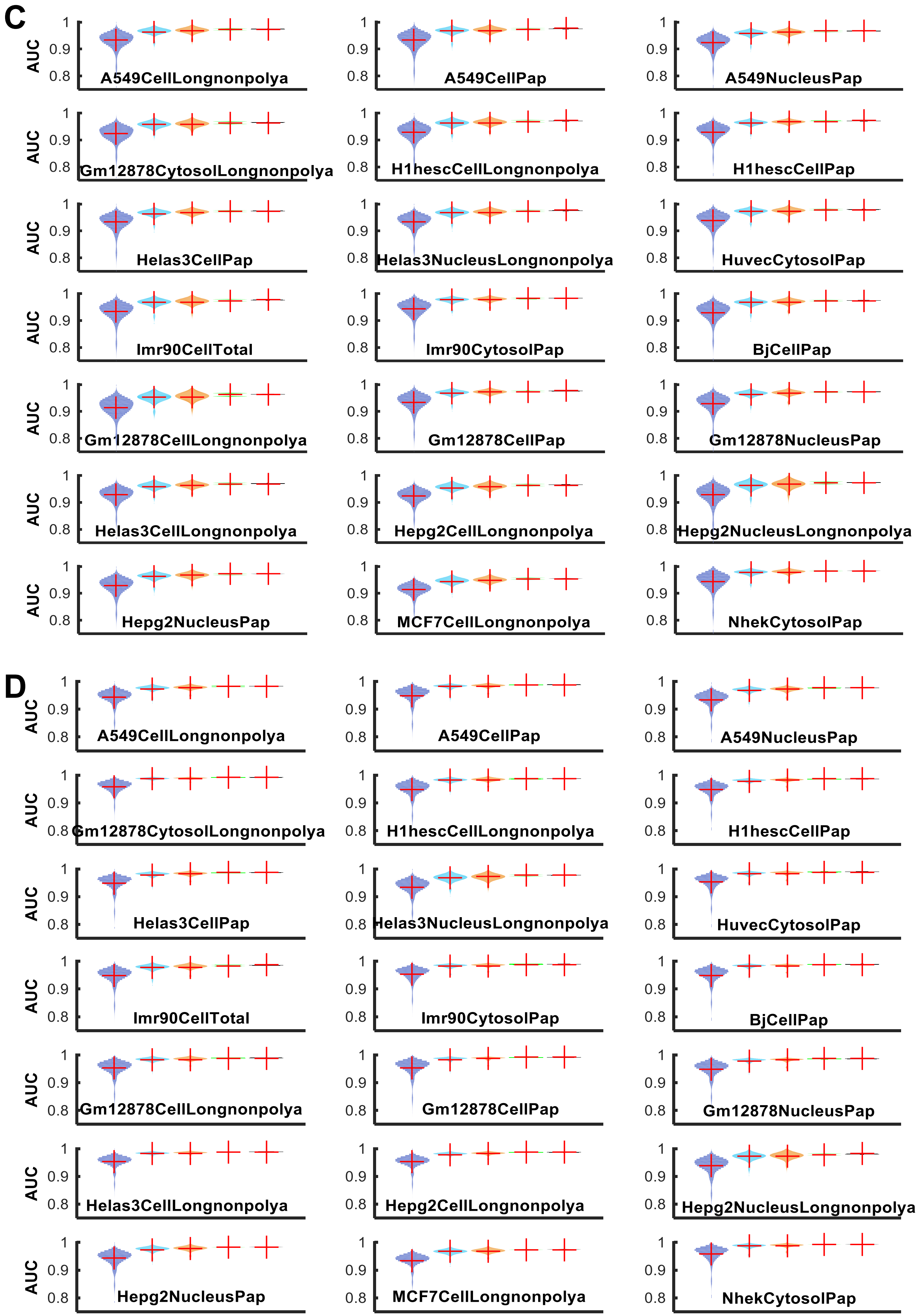
C
AUC
1
0.9
0.8
A549CellLongnonpolya
A549CellPap
A549NucleusPap
Gm12878CytosolLongnonpolya
H1hescCellLongnonpolya
H1hescCellPap
Helas3CellPap
Helas3NucleusLongnonpolya
HuvecCytosolPap
Imr90CellTotal
Imr90CytosolPap
BjCellPap
Gm12878CellLongnonpolya
Gm12878CellPap
Gm12878NucleusPap
Helas3CellLongnonpolya
Hepg2CellLongnonpolya
Hepg2NucleusLongnonpolya
Hepg2NucleusPap
MCF7CellLongnonpolya
NhekCytosolPap
D
AUC
1
0.9
0.8
A549CellLongnonpolya
A549CellPap
A549NucleusPap
Gm12878CytosolLongnonpolya
H1hescCellLongnonpolya
H1hescCellPap
Helas3CellPap
Helas3NucleusLongnonpolya
HuvecCytosolPap
Imr90CellTotal
Imr90CytosolPap
BjCellPap
Gm12878CellLongnonpolya
Gm12878CellPap
Gm12878NucleusPap
Helas3CellLongnonpolya
Hepg2CellLongnonpolya
Hepg2NucleusLongnonpolya
Hepg2NucleusPap
MCF7CellLongnonpolya
NhekCytosolPap

Fig. S9

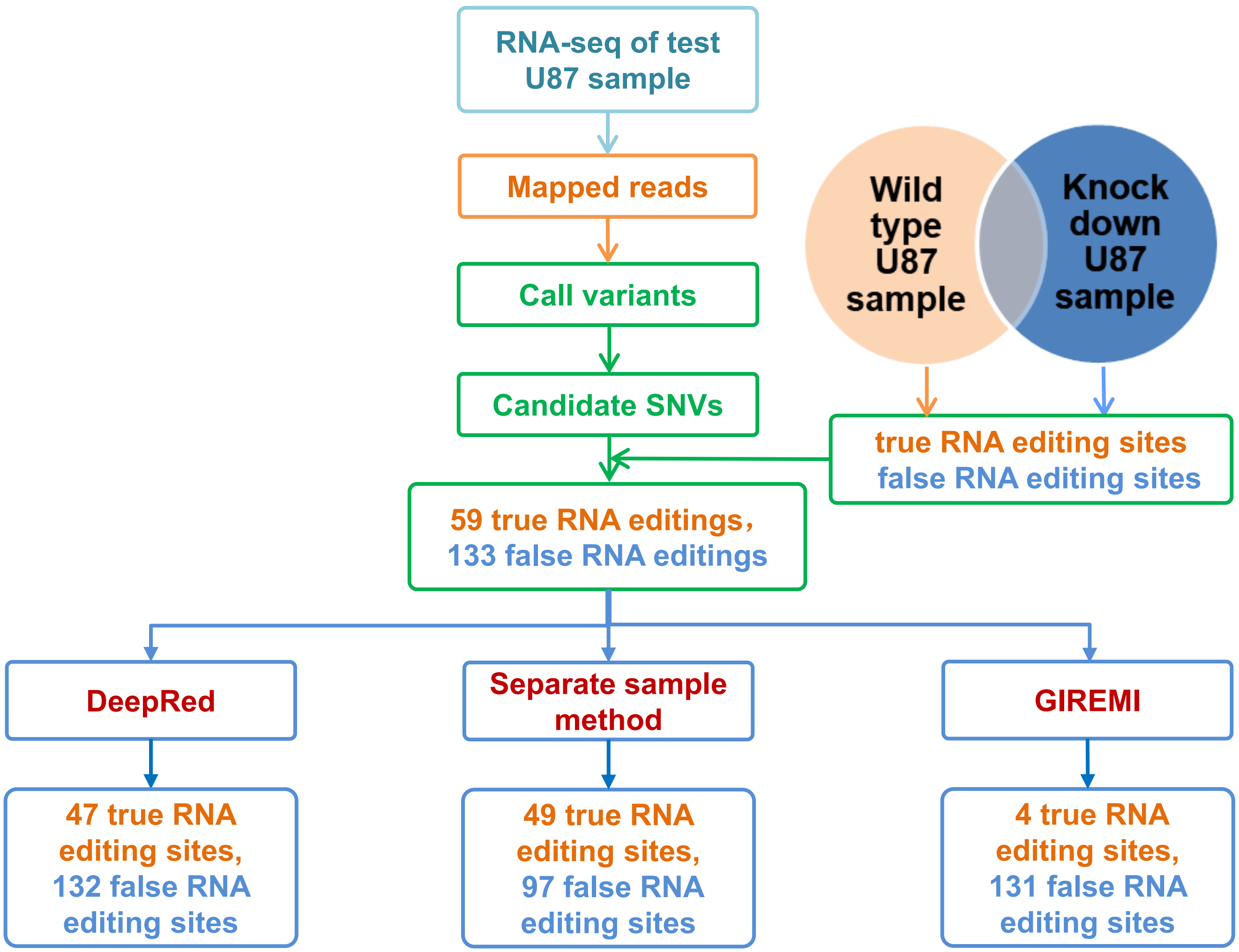
RNA-seq of test U87 sample
Mapped reads
Call variants
Candidate SNVs
Wild type U87 sample
Knock down U87 sample
true RNA editing sites
false RNA editing sites
59 true RNA editings,
133 false RNA editings
DeepRed
Separate sample method
GIREMI
47 true RNA editing sites,
132 false RNA editing sites
49 true RNA editing sites,
97 false RNA editing sites
4 true RNA editing sites,
131 false RNA editing sites

Fig. S10

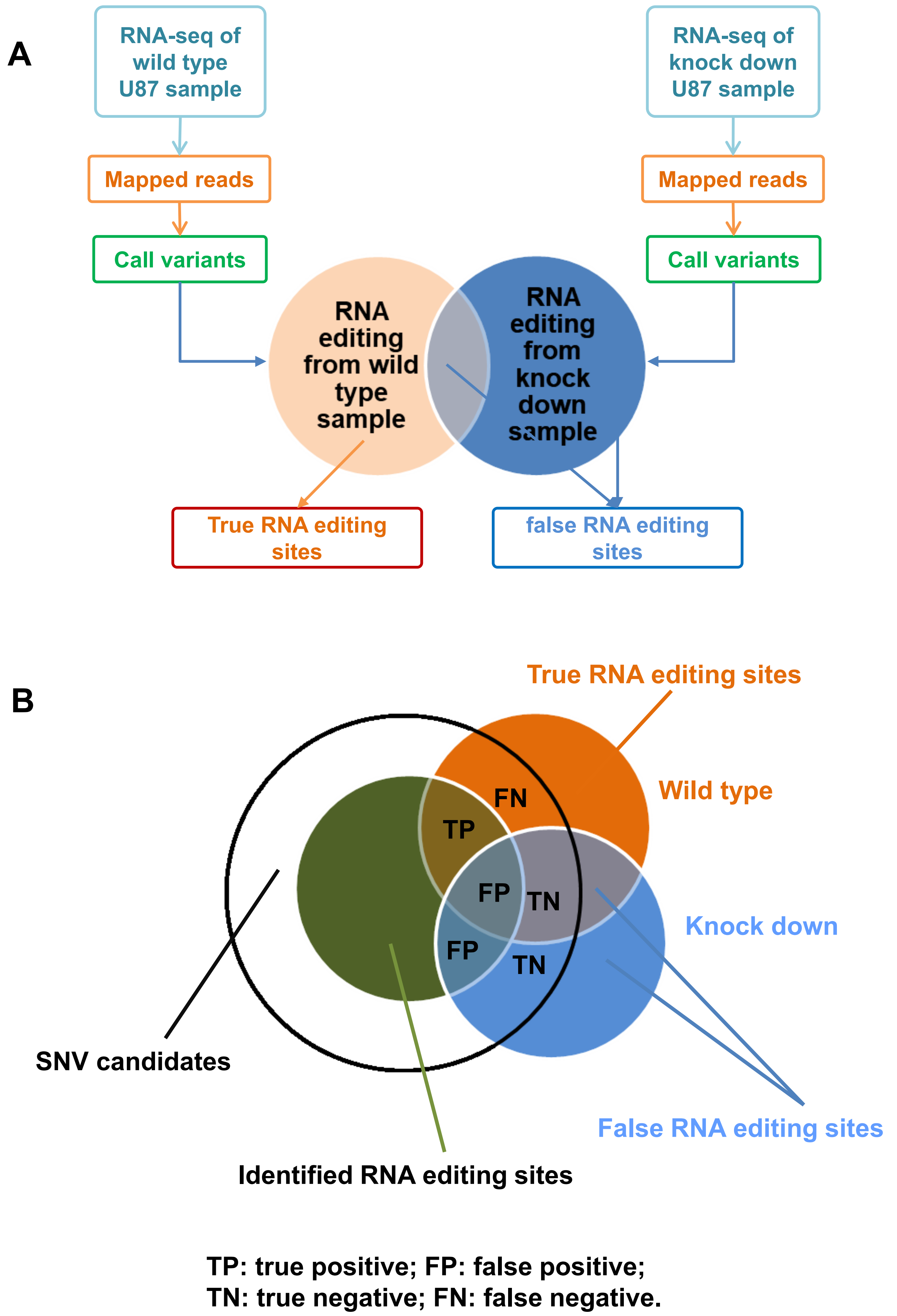
A
RNA-seq of wild type U87 sample
Mapped reads
Call variants
RNA-seq of knock down U87 sample
Mapped reads
Call variants
RNA editing from wild type sample
RNA editing from knock down sample
True RNA editing sites
false RNA editing sites
B
True RNA editing sites
Wild type
FN
TP
FP
TN
FP
TN
Knock down
SNV candidates
False RNA editing sites
Identified RNA editing sites
TP: true positive; FP: false positive;
TN: true negative; FN: false negative.

Fig. S11

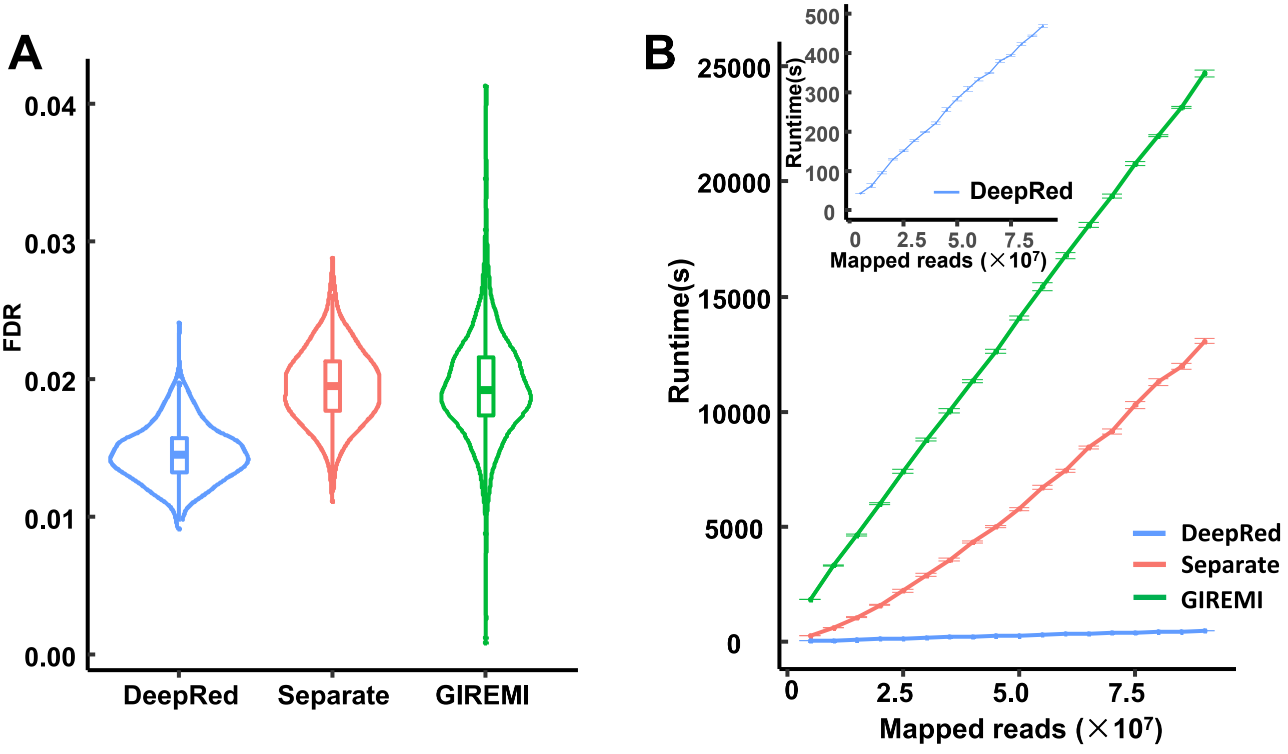
A
0.04
0.03
0.02
0.01
0.00
FDR
DeepRed
Separate
GIREMI
B
25000
20000
15000
10000
5000
0
Runtime(s)
0
2.5
5.0
7.5
Mapped reads (×10^7)
DeepRed
Separate
GIREMI
500
400
300
200
100
0
Runtime(s)
DeepRed
0
2.5
5.0
7.5
Mapped reads (×10^7)

Fig. S12

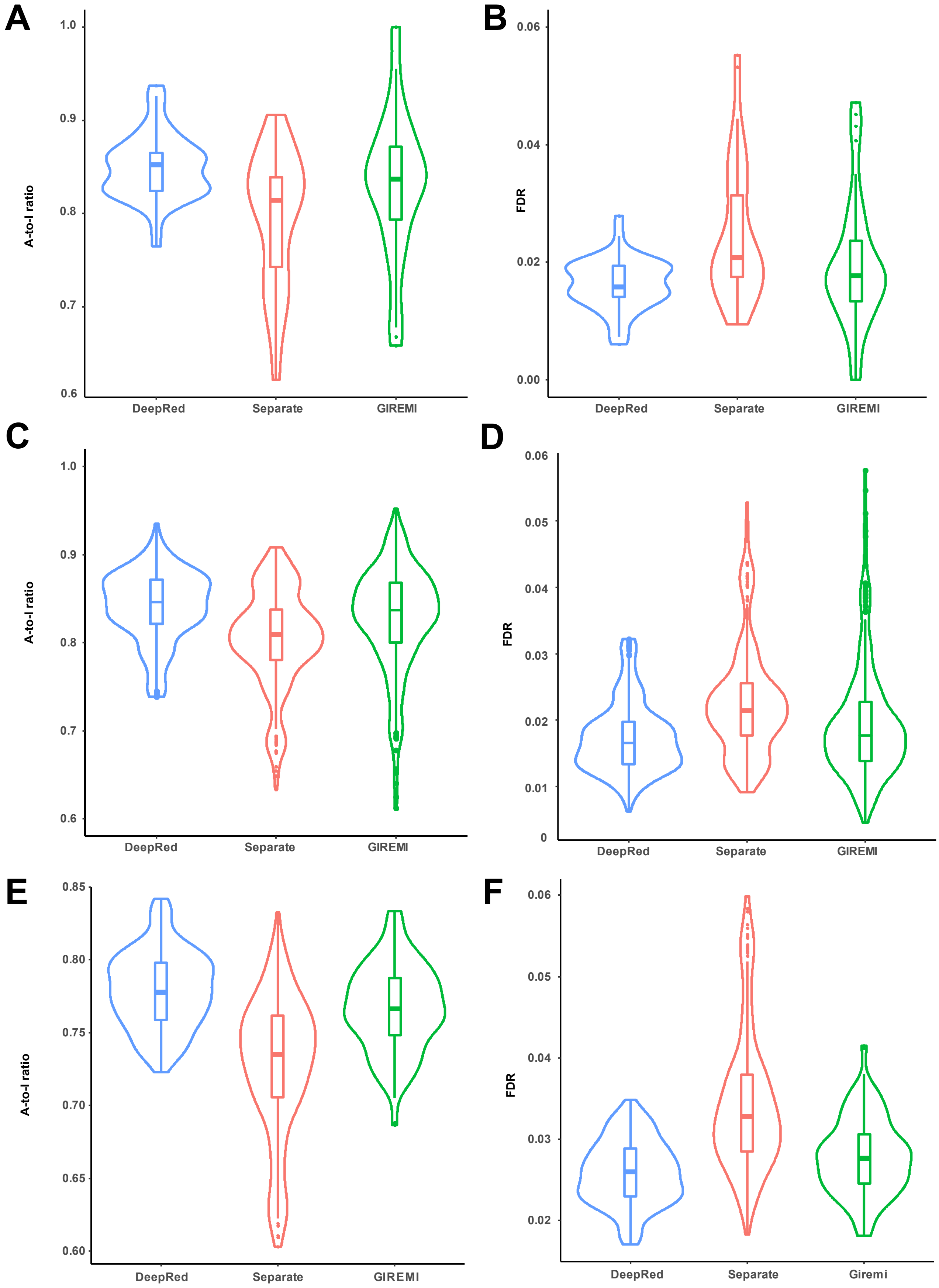
A
A-to-I ratio
1.0
0.9
0.8
0.7
0.6
DeepRed
Separate
GIREMI
B
FDR
0.06
0.04
0.02
0.00
DeepRed
Separate
GIREMI
C
A-to-I ratio
1.0
0.9
0.8
0.7
0.6
DeepRed
Separate
GIREMI
D
FDR
0.06
0.05
0.04
0.03
0.02
0.01
0
DeepRed
Separate
GIREMI
E
A-to-I ratio
0.85
0.80
0.75
0.70
0.65
0.60
DeepRed
Separate
GIREMI
F
FDR
0.06
0.05
0.04
0.03
0.02
DeepRed
Separate
Giremi

Fig. S13

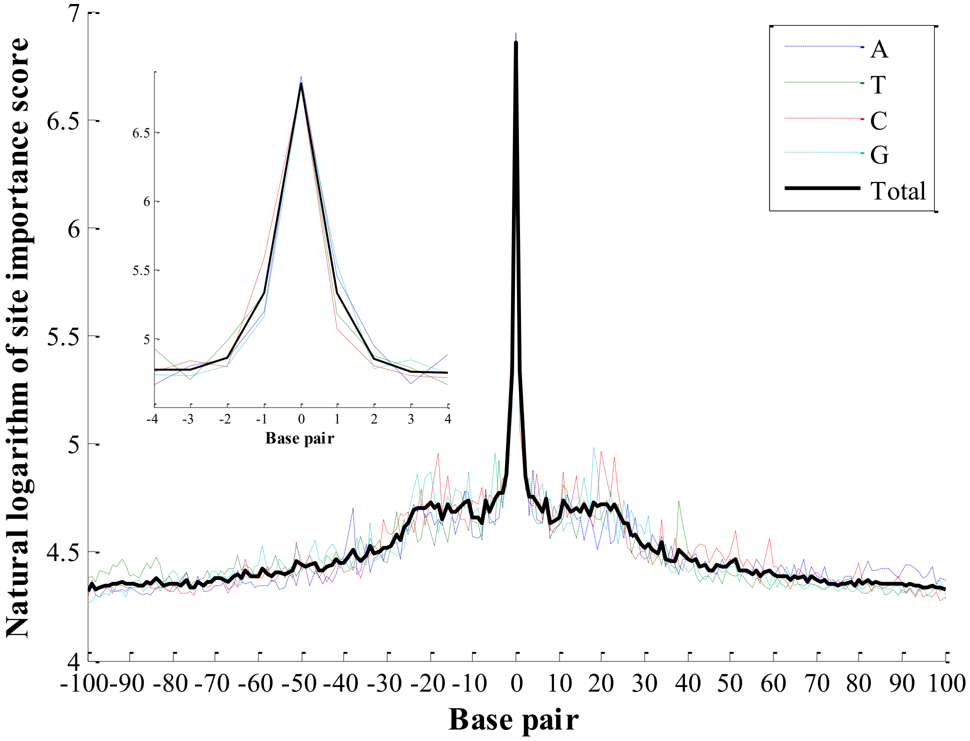
Natural logarithm of site importance score
7
6.5
6
5.5
5
4.5
4
-100 -90 -80 -70 -60 -50 -40 -30 -20 -10 0 10 20 30 40 50 60 70 80 90 100
Base pair
A
T
C
G
Total
6.5
6
5.5
5
-4 -3 -2 -1 0 1 2 3 4
Base pair

Fig. S14

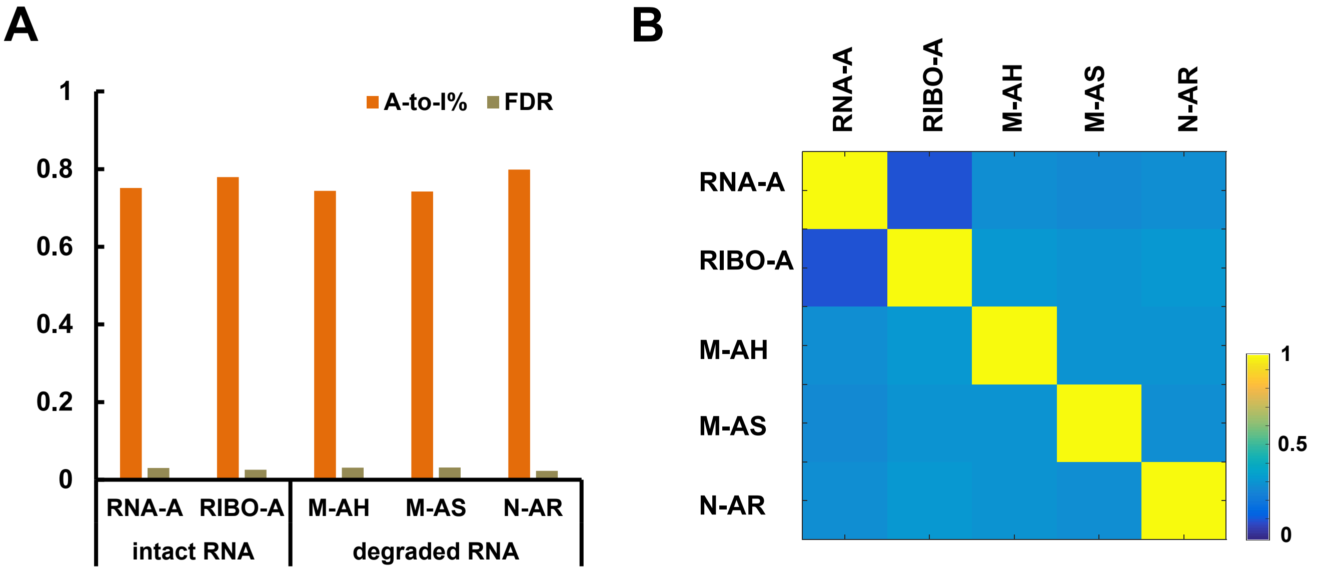
A
1
0.8
0.6
0.4
0.2
0
A-to-I%
FDR
RNA-A
RIBO-A
M-AH
M-AS
N-AR
intact RNA
degraded RNA
B
RNA-A
RIBO-A
M-AH
M-AS
N-AR
RNA-A
RIBO-A
M-AH
M-AS
N-AR
1
0.5
0

Fig. S15

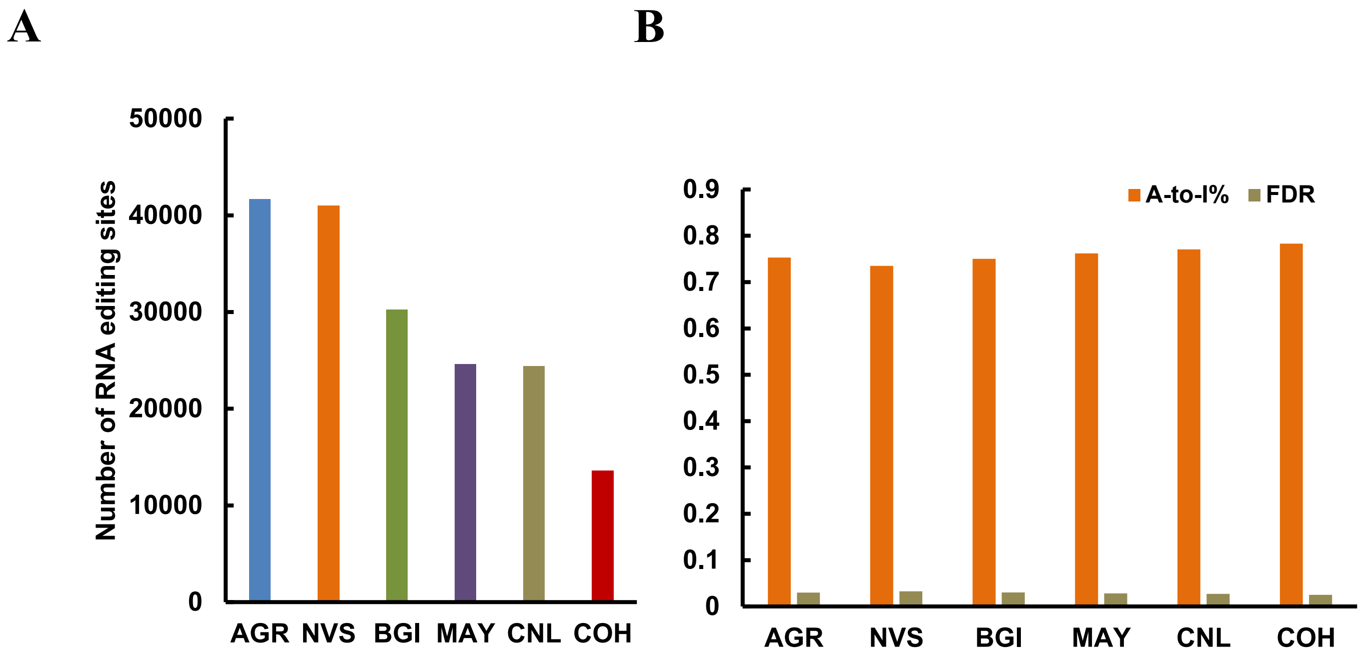
A
B
Number of RNA editing sites
50000
40000
30000
20000
10000
0
AGR
NVS
BGI
MAY
CNL
COH
0.9
0.8
0.7
0.6
0.5
0.4
0.3
0.2
0.1
0
A-to-I%
FDR
AGR
NVS
BGI
MAY
CNL
COH

Fig. S16

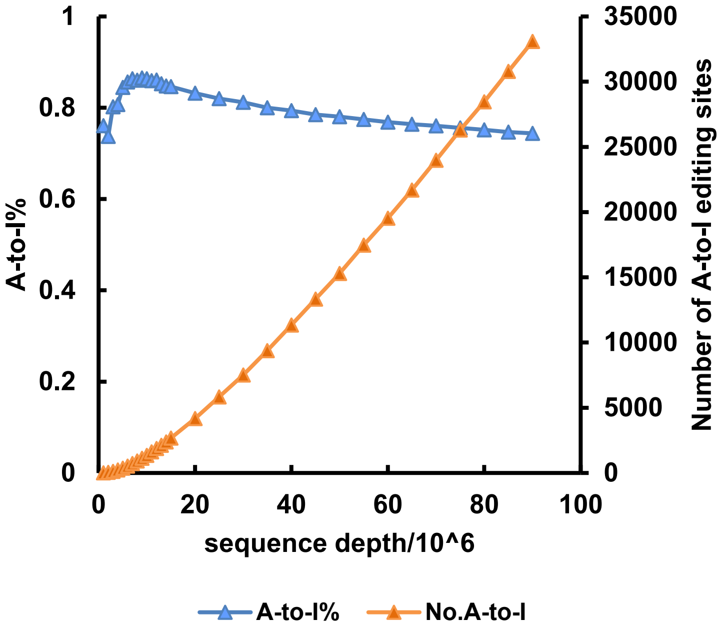
1
0.8
0.6
0.4
0.2
0
A-to-I%
35000
30000
25000
20000
15000
10000
5000
0
Number of A-to-I editing sites
0
20
40
60
80
100
sequence depth/10^6
A-to-I%
No.A-to-I

Fig. S17

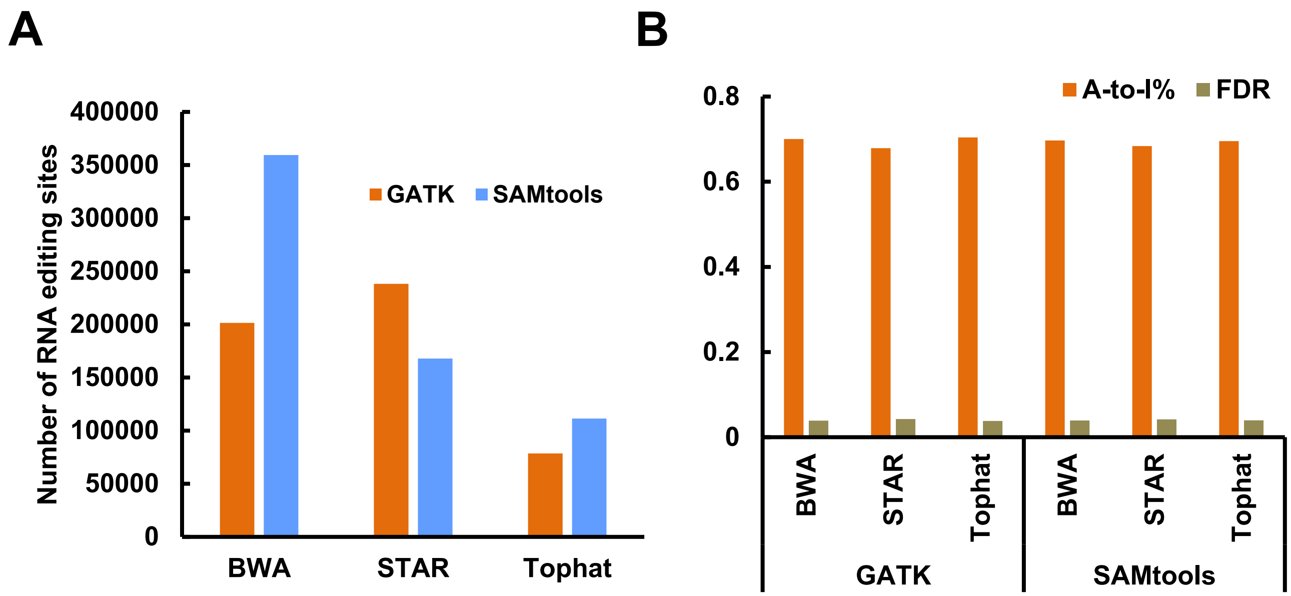
A
Number of RNA editing sites
400000
350000
300000
250000
200000
150000
100000
50000
0
GATK
SAMtools
BWA
STAR
Tophat
B
0.8
0.6
0.4
0.2
0
A-to-I%
FDR
BWA
STAR
Tophat
BWA
STAR
Tophat
GATK
SAMtools

Fig. S18

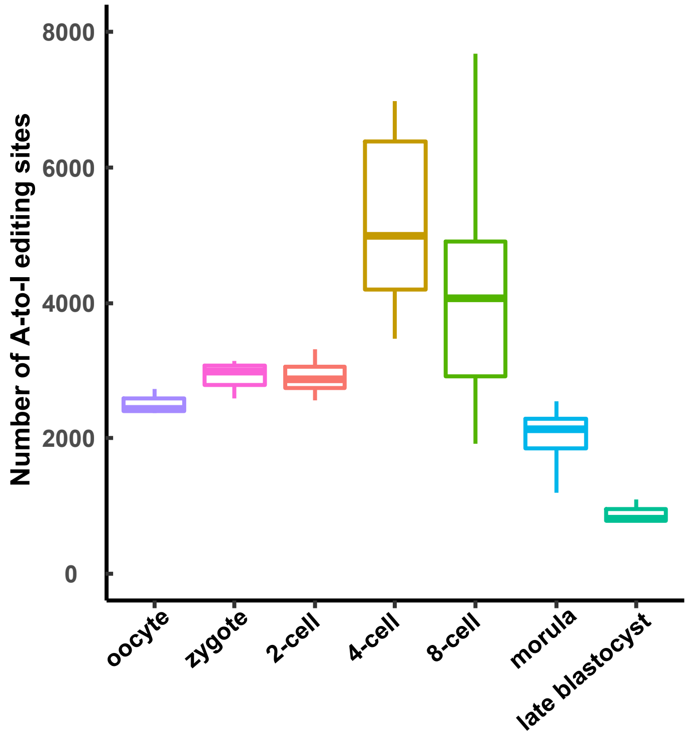

Number of A-to-I editing sites
8000
6000
4000
2000
0
oocyte
zygote
2-cell
4-cell
8-cell
morula
late blastocyst

Fig. S19

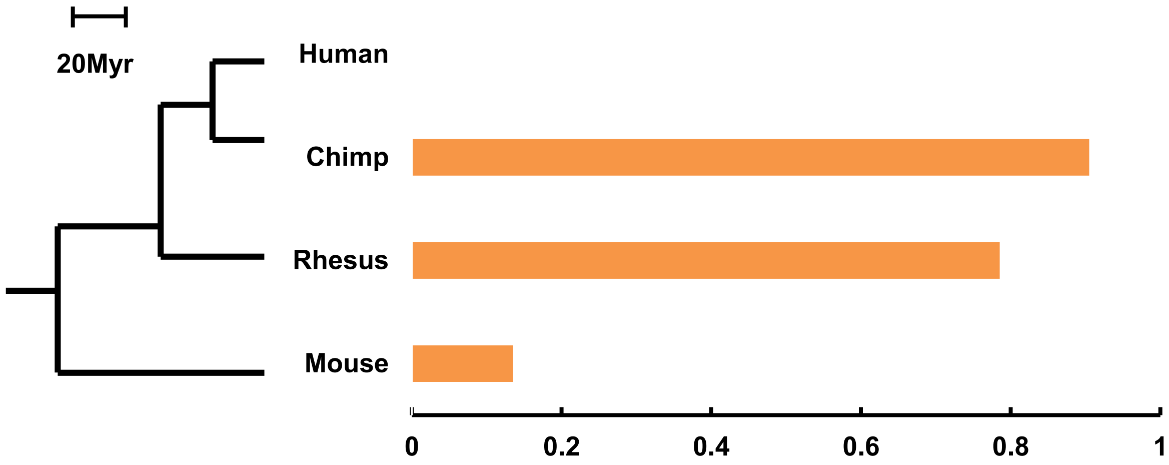

Fig. S20

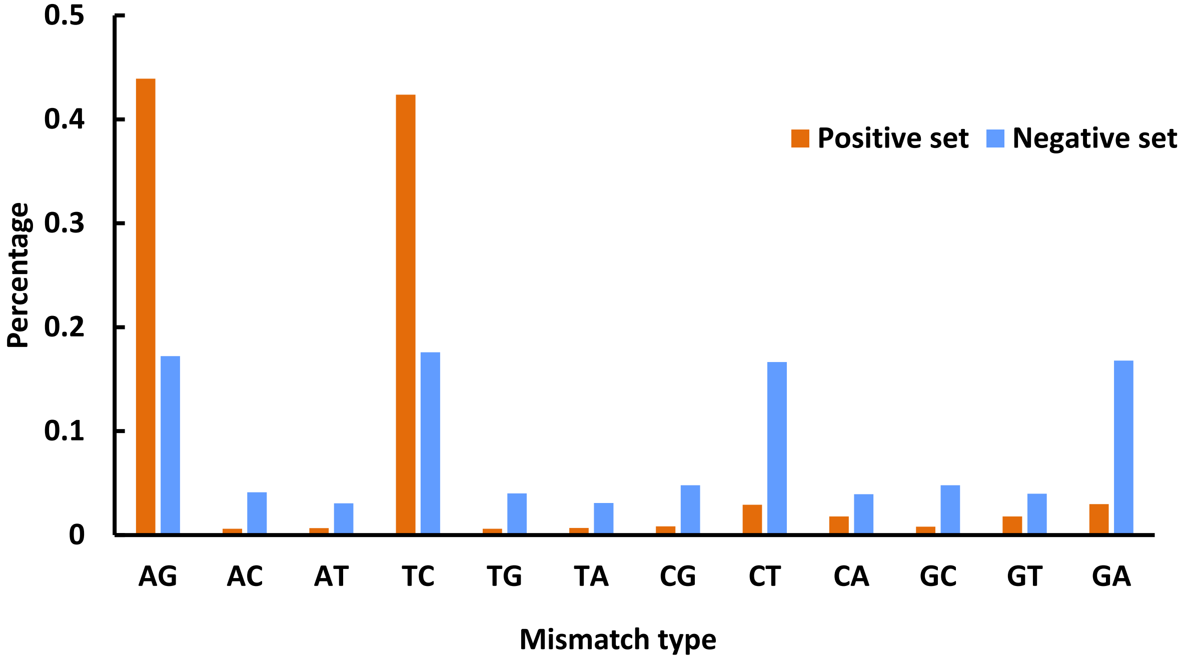
0.5
0.4
0.3
0.2
0.1
0
Percentage
Positive set
Negative set
AG
AC
AT
TC
TG
TA
CG
CT
CA
GC
GT
GA
Mismatch type

Fig. S21
